# Role of autobiographical memory in patient response to cognitive behavioural therapies for depression: protocol of an individual patient data meta-analysis

**DOI:** 10.1136/bmjopen-2019-031110

**Published:** 2019-06-12

**Authors:** Caitlin Hitchcock, Judita Rudokaite, Shivam Patel, Alicia Smith, Isla Kuhn, Edward Watkins, Tim Dalgleish

**Affiliations:** 1 MRC Cognition and Brain Sciences Unit, University of Cambridge, Cambridge, UK; 2 School of Clinical Medicine, University of Cambridge, Cambridge, UK; 3 Psychology, University of Exeter, Exeter, UK

**Keywords:** mental health, adult psychiatry

## Abstract

**Introduction:**

Cognitive behavioural therapies (CBTs) are one of the most effective treatments for major depression. However, ~50% of individuals do not adequately respond to intervention and of those who do remit from a depressive episode, over 50% will experience later relapse. Identification of patient-level factors which moderate treatment response may ultimately help to identify cognitive barriers that could be targeted to improve treatment efficacy. This individual patient data meta-analysis explores one such potential moderator—the ability to retrieve specific, detailed memories of the autobiographical past—as cognitive-based therapeutic techniques draw heavily on the ability to use specific autobiographical information to challenge the dysfunctional beliefs which drive depression.

**Methods and analysis:**

We have formed a collaborative network which will contribute known datasets. This will be supplemented by datasets identified through literature searches in Medline, PsycInfo, Web of Science, the Cochrane Central Register of Controlled Trials and WHO trials database between December 2018 and February 2019. Inclusion criteria are delivery of a cognitive or cognitive behavioural therapy for major depression, and measurement of autobiographical memory retrieval at preintervention. Primary outcomes are depressive symptoms and clinician-rated diagnostic status at postintervention, along with autobiographical memory specificity at postintervention. Secondary outcomes will consider each of these variables at follow-up. All analyses will be completed using random-effects models employing restricted maximum likelihood estimation. Risk of bias in included studies will be measured using the Revised Cochrane Risk of Bias Tool.

**Ethics and dissemination:**

The findings will be published in a peer-reviewed journal. Study results will contribute to better understanding of the role of autobiographical memory in patient response to CBTs, and may help to inform personalised medicine approaches to treatment of depression.

**PROSPERO registration number:**

CRD42018109673.

**Strengths and limitations of this study**

- This is the first individual patient data meta-analysis of the role of autobiographical memory in patient response to cognitive behavioural therapy (CBT) for major depression.
- This study may identify a patient-level moderator of response to CBTs.
- Results will advance understanding of whether CBTs improve this relapse risk factor.
- We anticipate that there may only be a small number of trials which have measured autobiographical memory, which may limit conclusions.

## Introduction

Depression is the second leading cause of disability worldwide.^1^ The disorder costs the UK economy an estimated £7.5 billion per year, with this cost expected to increase.^2^ Antidepressant medications (eg, selective serotonin reuptake inhibitors; tricyclic antidepressants) are effective in treating depression,^3 4^ but only so long as they are taken.^5 6^ Despite efficacious psychological treatments such as interpersonal psychotherapy^7 8^ and cognitive behavioural therapies (CBTs),^9 10^ only 40%–60% of National Health Service (NHS) patients experience recovery after receiving psychological intervention for depression.^11^ Of those who do respond, up to 50% will experience later relapse.^12^ Our gold-standard psychological interventions are therefore producing suboptimal outcomes for a significant number of individuals.

Improving treatment success rates is therefore a key priority for social, economic and health systems. Identification of patient-level factors which may interfere with the efficacy of psychological interventions and predispose an individual towards poorer treatment response may help to explain differential response rates. If we consider CBTs, which are commonly administered in front-line NHS psychology services (eg, improving access to psychological therapies services),^11^ identification of cognitive barriers which may impede the ability to complete cognitive therapy tasks may advance our understanding of exactly why some individuals respond poorly, or not at all. Similarly, cognitive predictors of depression which are not shifted by intervention may indicate relapse risk factors.^13^ Together, further understanding of patient-level factors that both impede treatment efficacy and/or are not shifted by treatment and go on to predict relapse may contribute to development of a personalised medicine approach^14^ for treatment of depression.

Autobiographical memory, our memory for personal life experiences, is one cognitive factor which plays an important role in the course of depression.^15^ In particular, the ability to retrieve specific, detailed memories of single incidents (eg, *the occasion I met my partner*) predicts future depressive symptoms over and above current symptom levels (for a meta-analysis, see Sumner *et al*).^16^ The ability to retrieve specific autobiographical memories not only independently predicts prognosis (eg, by reducing the ability to solve problems or plan for the future^17 18^) but may also impede the ability to complete CBT for depression.

A core aim of both traditional and later wave CBTs is to change the way that an individual relates to dysfunctional thoughts about the self and world (originally outlined by Beck).^19^ Key therapeutic techniques encourage the individual to identify links between thoughts and feelings, by recalling what they said to themselves, how that made them feel and how that led them to act in a recent situation (eg, *going to a work function*). For example, a core technique is to encourage the individual to not automatically accept thoughts as facts, but rather, to evaluate the accuracy of each thought (eg, *no one wants to talk to me*) using specific pieces of evidence recalled from the individual’s past (eg, *last time I was at a work function I had a lovely conversation with a colleague*). The efficacy of such techniques is therefore likely to be influenced by an individual’s ability to recall detailed, specific information about their autobiographical past. Treatment manuals instruct cognitive therapists to enrich detail and encourage specificity in patient responses while they are completing such tasks; however, a moderating role of memory specificity in the efficacy of CBTs has yet to be explored.

Similarly, it is possible that this reduction in autobiographical memory specificity may also be improved as a result of completing CBT. In continuously promoting a focus on specific information, and reducing the depressive tendency to overgeneralise information, CBT techniques may actively train an individual to be more specific. Indeed, increasing the specificity of thinking has been previously proposed as a key mechanism of effective CBT for depression.^20^ There is some prior literature which suggests that autobiographical memory specificity may improve following a course of cognitive-based intervention, most notably CBT^21^ and mindfulness-based cognitive therapy.^22^ In contrast, evidence that reduced memory specificity persists between depressive episodes^16^ may indicate that this relapse risk factor is not commonly shifted by treatment. Determining the impact of CBTs on autobiographical memory specificity, relative to natural recovery, antidepressant medication and other active psychological interventions, will advance our understanding of how treatment impacts this relapse risk factor, and offer important implications for the adjunctive use of autobiographical memory-based interventions (for review, see Barry *et al* 23; Hitchcock *et al*)^24^ to enhance treatment outcomes.

The lack of prior investigation of the interaction between autobiographical memory specificity and CBTs has largely been due to a lack of statistical power to examine moderation effects in prior studies. Prior trials of CBTs which have included a measure of autobiographical memory have recruited small sample sizes (eg, n=21 in the Mindfulness Based Cognitive Therapy (MBCT) group in Williams *et al*),^22^ and thus been underpowered to explore these effects. Completion of individual patient data meta-analysis (IPD-MA) can overcome this issue. The IPD-MA approach involves synthesis of patient-level data across multiple studies, thereby offering greater statistical potential to explore individual characteristics and how these interact with treatment effects, relative to aggregate meta-analysis which synthesises data at the study level.^25^ We have therefore formed a collaborative network to allow us to complete an IPD-MA evaluating the nature of autobiographical memory specificity in patient response to cognitive behavioural treatment for depression.

Specifically, we will address the following questions:

1. First, does treatment for depression (a) induce a change in autobiographical memory specificity? and (b) induce a stronger change following CBTs relative to other interventions?
2. Second, does the specificity of autobiographical memory at baseline predict treatment response (a) for all interventions (predictor effect); and (b) is this effect different for CBTs relative to other interventions (moderation effect)?

## Method

### Study registration and management

This IPD-MA will be conducted in accordance with Preferred Reporting Items for Systematic Reviews and Meta-Analyses guidelines,^26^ and is registered on PROSPERO. Regular email updates will be used to inform the collaborating network of our activities. Encrypted electronic data sharing clouds and email will be used to exchange data and paperwork between researchers.

### Criteria for included studies

#### Types of studies

Included studies will be both controlled and uncontrolled clinical trials. Studies may be randomised at patient level, cluster-randomised or non-randomised. For crossover design studies, only data for the initial phase (ie, pre crossover) will be used. Articles must be published in English. Unpublished data will be actively sought; hence, non-peer-reviewed studies will also be included. Sensitivity analyses will be completed to evaluate the impact of study type on our results.

#### Participants

Studies must have recruited participants aged 18 years or older. Participants must have a diagnosis of major depressive disorder (MDD) according to the Diagnostic and Statistical Manual of Mental Disorders (any edition) or International Classification of Diseases (any edition) criteria, assessed via a structured clinical interview (eg, Structured Clinical Interview for DSM Disorders; Mini International Neuropsychiatric Interview). As depression-related deficits in autobiographical memory are evident into remission^16^ and National Institute of Health and Care Excellence guidelines recommend that CBT interventions are used to reduce depressive relapse (eg, MBCT),^27^ participants may either be experiencing a current major depressive episode, or remitted from MDD.

#### Autobiographical memory

Studies must include an objective measure of the specificity of autobiographical memory. This may be indexed using a cued-recalled task (eg, the autobiographical memory task),^28^ an interview^29^ or sentence completion procedure^30^ but responses must be scored for the number of specific, single incident memories retrieved.

#### Intervention

We will include studies evaluating any psychological therapy for depression which is cognitively based as per the Beck model (eg, cognitive therapy, CBT, mindfulness-based cognitive therapy). This will be determined by two independent reviewers using the author’s intervention description. Interventions may be self-guided or therapist-guided, and delivered in-person or online, and in individual or group-based format. Excluded interventions will be those which directly aim to improve a defined cognitive process or bias (eg, cognitive/attention/interpretative bias modification, autobiographical memory-based intervention, concreteness training).

#### Comparison condition

Studies may include no comparison condition (ie, uncontrolled trials) or may include any type of comparison condition. Comparison conditions are anticipated to be waitlist, any psychosocial intervention (eg, psychoeducation, counselling) or antidepressant medication. Again, condition type will be determined by two independent reviewers based on the author’s intervention description. CBTs will be compared (1) against waitlist control (to index effects against natural recovery), (2) against all other interventions, (3) against other psychological interventions alone and (4) against antidepressant medication alone.

#### Coprimary outcomes

For question 1, the primary outcome will be autobiographical memory specificity at postintervention. This will be operationalised as the proportion of specific, single incident memories reported by the participant during an objective measure of autobiographical memory. Specificity of memories will be rated by researchers (not participants). Proportions (number specific/number of trials) will be calculated for any studies reporting the number of specific memories.

For question 2, the coprimary outcomes will be treatment response at postintervention, indexed as (1) a score on a continuous measure of depressive symptoms and (2) diagnostic status, as rated using a standardised clinician-administered interview. In situations where more than one continuous measure of depression is administered, the Beck Depression Inventory II (BDI-II) will be preferred in analysis as this has been specifically devised to measure changes following CBT interventions. Any studies reporting Beck Depression Inventory I scores will be converted to BDI-II scores using the method outlined in the BDI-II manual.^31^

#### Secondary outcomes

As it is highly desirable for an intervention to produce stable, lasting change, follow-up assessments will be considered as secondary outcomes. The secondary outcomes for question 1 will be autobiographical memory specificity (as per defined above) at follow-up assessments. The secondary outcome for question 2 will be depressive symptoms and diagnostic status (as per defined above) at follow-up assessments.

Follow-up lengths to be included are assessments between 1 month and 2 years following the completion of therapy. During analysis, studies including a follow-up assessment between 1 and 3 months postintervention will be grouped to form a short-term follow-up, and any later assessment points will be grouped per 6-month period (ie, 6 and 12 months). This will result in analysis of follow-up outcomes in the short-term (1–3 months), and at 6, 12, 18 and 24 months postintervention.

#### Moderator

For question 2, the moderating variable will be the proportion of specific, single incident memories reported by the participant during an objective measure of autobiographical memory at baseline.

### Search methods for identification of studies

#### Collaborative network

We have formed a collaborative network of experts in the role of autobiographical memory in depression. Through our own work and knowledge of the field, we have initially identified and obtained access to four individual datasets which provide the data necessary to analyse our research questions. Using this network to identify other potential datasets for inclusion, we will email the autobiographical memory and psychopathology special interest group, and the collaborators of each member of our network. We will also make use of social media such as Twitter to raise awareness of the IPD-MA. This mode of identification is important, as we anticipate that many of the trials which have included an autobiographical memory measure may not list the measure in the Method section of the published paper. Indeed, this is the case for the majority of the datasets the collaborative network has already identified.

#### Electronic searches

**Figure 1:**
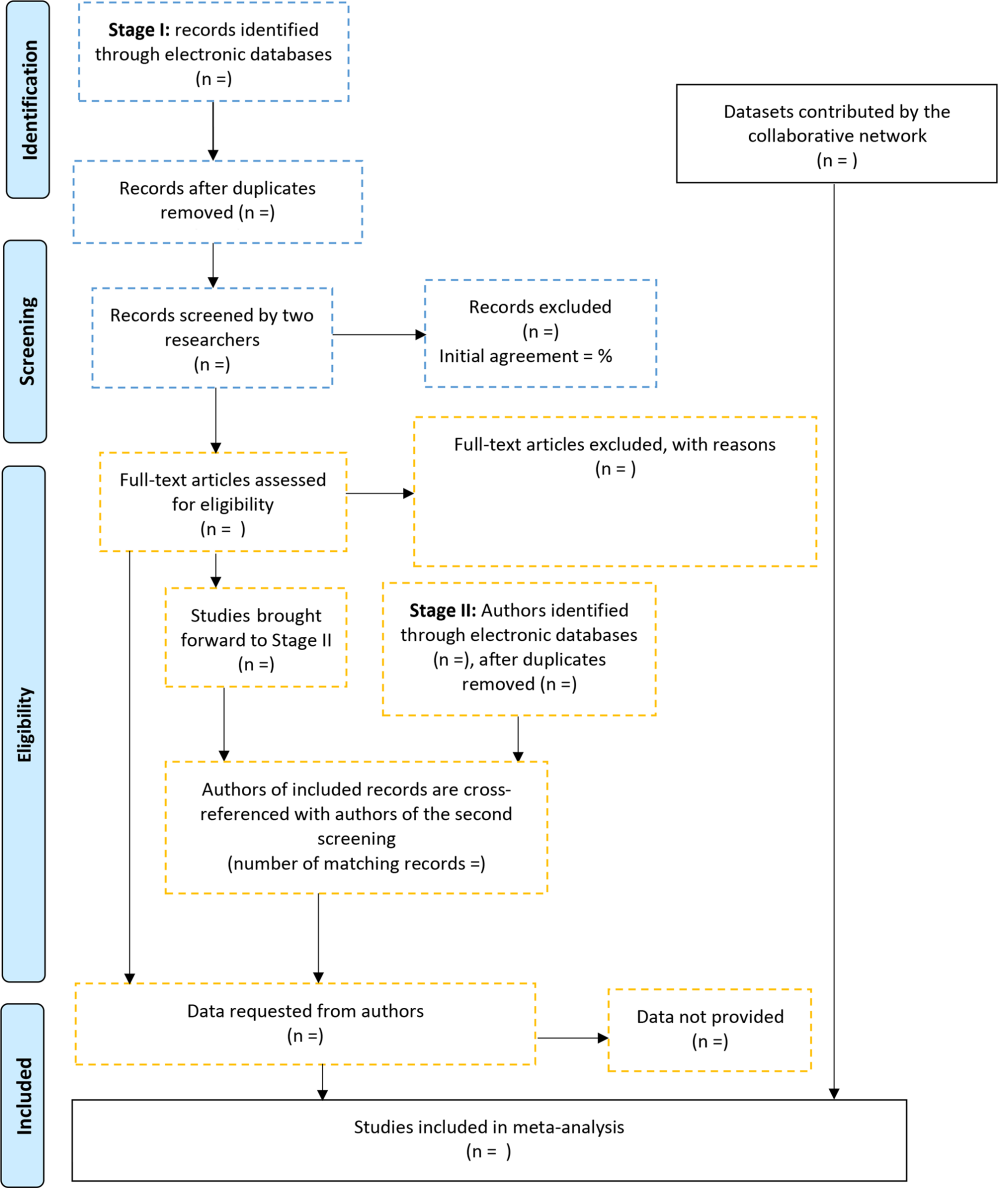
Identification of eligible datasets.

As we anticipate that many of the trials which have included an autobiographical memory measure may not list this measure in the published paper, a multistage search strategy will be completed in order to identify all potential datasets (see figure 1).

First, searches will be completed in PsycINFO, Medline, Web of Science, Cochrane database and WHO trials database, using the string: cognitive therapy AND depression AND trial AND any flag words (with OR inserted between every flag word*) we have identified as markers of papers which may potentially include an autobiographical memory measure. These flag words were identified by evaluating common terminology in the abstracts of the four datasets identified through the collaboration network. These terms are ‘mechanism,’ ‘process’, ‘memory’, ‘cognitive function’, ‘negative thinking’, ‘rumination’, ‘depressogenic thinking’ and ‘autobiographical memory’. We use the term ‘cognitive therapy’ although we will also include CBTs as use of the term ‘cognitive behavioural therapy’ may miss studies which administer cognitive therapy. Conversely, searches using the term cognitive therapy did identify evaluations of CBTs. Searches will be restricted to papers published in or after 1986 as this was the year that the seminal paper on autobiographical memory specificity in depression was published.^28^ The full electronic search strategy is available in the online supplementary materials.

10.1136/bmjopen-2019-031110.supp1 Supplementary data

Search results will be imported into Rayyan (a web-based tool for managing systematic reviews) by an administrator (Kuhn) and duplicates will be removed. This will allow for blind screening by two independent reviewers (Rudokaite and Patel). Papers deemed ineligible from title and abstract by both reviewers will be set aside. Full text of the remaining papers will be examined independently by the reviewers to determine eligibility. Any discrepancy in inclusion will be resolved via discussion with Hitchcock, or if necessary, by contacting the study authors for clarification.

Next, a secondary screening will be completed in order to identify studies which may have measured autobiographical memory but not reported on it in the published paper. Through searches in Medline, PsycINFO and Web of Science using the terms autobiographical memory AND depression, we will produce a list of authors who have ever published on autobiographical memory and depression. This list will then be cross-referenced with the results of the primary database search. For studies with an author who has previously published on autobiographical memory, corresponding authors will then be emailed to enquire about unpublished autobiographical memory data.

The electronic search was conducted between December 2018 and February 2019.

### Data collection

Corresponding authors of eligible studies will be emailed to request data. A reminder email will be sent every 2 weeks. If an author does not respond after 1 month, another author of the study will be contacted. A second attempt to contact this author will follow and so forth until a maximum of three authors are contacted. Study data will be considered unavailable if no study authors have responded to multiple contact attempts, or if authors indicate that they no longer have access to the data.

The authors will be able to supply data via email or an encrypted online data sharing service operated by the University of Cambridge. A single author will be identified for each study to whom all queries about the data collection processes and transformation of individual variables will be addressed. When researchers are cleaning a specific data set they may communicate with the original investigators via telephone discussions or by email.

### Data extraction, quality checks and storage

In accordance with Cochrane recommendations, both published and unpublished measures will be collected for all studies. IPD for specified variables (presented in table 1) will be extracted into a single dataset. For all outcomes, unimputed and untransformed data will be preferred. Spot checks will be completed to ensure data quality. The pattern of treatment allocation for each included study will be checked to ensure that blinding, randomisation and allocation sequence appear appropriate, in accordance with guidelines recommended by Tierney *et al*.^32^ Data will be stored in password-protected files on an encrypted University of Cambridge server, and will not include any personally identifiable information.

**Table 1:** Individual patient data to be extracted from included studies

Trial-level data	Patient characteristics	Outcomes
Trial identifier	Anonymised participant identifier	Post-treatment self-reported depressive symptoms
Country of completion	Age	Follow-up self-reported depressive symptoms
Type of CBT	Gender	Diagnostic status at post-treatment
Type of comparison group/s	Education history	Diagnostic status at follow-up
Format of treatment (individual, group)	Ethnicity	Post-treatment score for proportion of specific memories
Length of treatment in weeks	Concurrent treatment use (eg, medication)	Post-treatment score for proportion of non-specific memories
Information about risk of bias Trial design	Pre-treatment self-reported depressive symptoms	Follow-up score for proportion of specific memories
	Pre-treatment score for number of specific memories	Follow-up score for proportion of non-specific memories
	Pre-treatment score for number of non-specific memories	
	Any potential cognitive covariates (eg, rumination, working memory, IQ, executive function)	
	Reasons for missing data	

CBT, cognitive behavioural therapy; IQ, Intelligence Quotient.

### Risk of bias

For individual studies, risk of bias will be evaluated using the Revised Cochrane Risk of Bias Tool^33^ to access study quality and risk of bias due to the randomisation process, deviations from intended interventions, missing outcome data, measurement of the outcome, selection of the reported result and other biases such as conflicts of interest. Using the tool, each study will be rated as of high, low or unclear risk. In order to reduce attrition bias, data will be checked against trial Consolidated Standards of Reporting Trials diagrams to ensure that data from all randomised participants are included.

### Strategy for data synthesis

Intent-to-treat analysis will be completed using data imputed at within-study level (if missing at random assumptions are met). Between-study heterogeneity will be indexed using the tau statistic. As there are now multiple IPD-MAs establishing the effect of CBTs on depressive outcomes,^34 35^ we will proceed directly to evaluating moderation effects.

All analyses will be completed using random-effects models employing restricted maximum likelihood estimation. However, if there is considerable heterogeneity in the quality of studies (indexed by the Cochrane Risk of Bias Tool) sensitivity analysis will be completed using fixed-effects models such that poorer quality studies will not receive equal weighting. If IPD are available for more than seven studies, prediction intervals will be used.^36^ If we obtain sufficient power, subsets of studies will be analysed to explore whether the below effects vary based on trauma history (as trauma exposure may reduce memory specificity),^37^ depressive severity and type of therapy (eg, MBCT, CBT).

#### Question 1

The effect of intervention on autobiographical memory specificity will be analysed using an Analysis of Covariance (ANCOVA) random treatment effect one-stage model with random intercept and memory specificity baseline adjustment with different residual variance per study. Sensitivity analysis will be completed using stratified intercepts.

#### Question 2

To determine whether autobiographical memory specificity moderates treatment response, a two-stage model predicting post-treatment depressive symptoms with baseline symptom adjustment is planned to allow synthesis of datasets which used different symptom measures (eg, BDI-II and Hamilton Depression Rating Scale). The interaction term (baseline score x treatment type) will be estimated per trial, then pooled in a random-effects meta-analysis. Sensitivity analysis will next be completed using separate one-stage models for different depressive symptom measures. Patient-level covariates will be centred to separate within-trial and across-trial effects.

Effects on diagnostic status will be analysed using a one-stage logistic model with random intercept to account for correlation between the interaction estimate and other parameter estimates. Again, patient-level covariates will be centred to separate within-trial and across-trial effects. Sensitivity analysis will be completed using stratified intercepts. If the one-stage model fails to converge, a two-stage model will be completed.

### Ethics and dissemination

Contributing studies will be asked to provide evidence of local ethical committee approval and to demonstrate that informed consent has been given to share deidentifed data. Contributing studies will also be required to remove patient identifiers before providing their data. This includes date of birth, which will be converted to age at time of assessment. A data-sharing agreement will be signed between the authors of included studies and the IPD-MA research team. The resulting IPD-MA dataset will be shared through the MRC Cognition and Brain Sciences Unit Data Repository (accessed via www.mrc-cbu.cam.ac.uk/publications/opendata/) in accordance with open-science practices.

### Patient and public involvement

Members of the patient and public involvement group formed by individuals with lived experience of depression discussed the project and the role of specificity in CBTs, and will contribute to dissemination of results.

## Supplementary Material

Reviewer comments

Author's manuscript
