# Supplementary material for: Role of autobiographical memory in patient response to cognitive behavioural therapies for depression: protocol of an individual patient data meta-analysis
Source: BMJ Open. 2019 Jun 12;9(6):e031110. doi: 10.1136/bmjopen-2019-031110 (PMC6575822; doi:10.1136/bmjopen-2019-031110)

Overview – Stage 1: CBT & Depression – RCT - keywords

	Hits
Medline	1746
Web of Science	3994
PsycINFO	1189
Cochrane – Trials Database	4566
WHO	149
**Total**	**11637**
**Deduplication**	**8719**

No limits were applied in terms of pu

Medline

Database: Ovid MEDLINE(R) and Epub Ahead of Print, In-Process & Other Non-Indexed Citations, Daily and Versions(R) <1946 to November 5, 2018>
Search Strategy:
----
1 exp behavior therapy/ or (cognit* adj5 behav*).mp. or ((cognit* or behav* or conditioning or relaxation or desensiti*) adj5 (therap* or psychotherap* or train* or retrain* or treat* or modif*)).mp. or (implosive* adj3 therap*).mp.
2 exp depression/ or exp depressive disorder/ or depress*.mp.
3 (Randomized Controlled Trials as Topic/ or randomized controlled trial/ or Random Allocation/ or Double Blind Method/ or Single Blind Method/ or clinical trial/ or clinical trial, phase i.pt. or clinical trial, phase ii.pt. or clinical trial, phase iii.pt. or clinical trial, phase iv.pt. or controlled clinical trial.pt. or randomized controlled trial.pt. or multicenter study.pt. or clinical trial.pt. or exp Clinical Trials as topic/ or (clinical adj trial$).tw. or ((singl$ or doubl$ or treb$ or tripl$) adj (blind$3 or mask$3)).tw. or PLACEBOS/ or placebo$.tw. or randomly allocated.tw. or (allocated adj2 random$).tw.) not (case report.tw. or letter/ or historical article/)
4 (Mechanism* or process* or memor* or (cognitive adj function*) or ((negativ* or depressogenic*) adj3 think*) or ruminat*).mp.
5 1 and 2 and 3 and 4

Web of Science

5 #4 AND #3 AND #2 AND #1
Indexes=SCI-EXPANDED, SSCI, A&HCI, CPCI-S, CPCI-SSH, BKCI-S, BKCI-SSH, ESCI, CCR-EXPANDED, IC Timespan=All years

4 **TOPIC:** ((Mechanism* or process* or memor* or (cognitive near/1 function*) or ((negativ* or depressogenic*) near/3 think*) or ruminat*))
Indexes=SCI-EXPANDED, SSCI, A&HCI, CPCI-S, CPCI-SSH, BKCI-S, BKCI-SSH, ESCI, CCR-EXPANDED, IC Timespan=All years

3 **TOPIC:** (trial* or ((singl* or doubl* or trebl* or tripl*) near/1 (blind* or mask*)) or random*)
Indexes=SCI-EXPANDED, SSCI, A&HCI, CPCI-S, CPCI-SSH, BKCI-S, BKCI-SSH, ESCI, CCR-EXPANDED, IC Timespan=All years

2 **TOPIC:** (depress*)
Indexes=SCI-EXPANDED, SSCI, A&HCI, CPCI-S, CPCI-SSH, BKCI-S, BKCI-SSH, ESCI, CCR-EXPANDED, IC Timespan=All years

1 **TOPIC:** ((cognit* near/5 behav*) or ((cognit* or behav* or conditioning or relaxation

or desensiti*) near/5 (therap* or psychotherap* or train* or retrain* or treat* or modif*)) or (implosive* near/3 therap*))
Indexes=SCI-EXPANDED, SSCI, A&HCI, CPCI-S, CPCI-SSH, BKCI-S, BKCI-SSH, ESCI, CCR-EXPANDED, IC Timespan=All years

PsycINO

#	Query	Results
S12	S4 AND S7 AND S8 AND S11	1,189
S11	S9 OR S10	87,580
S10	(clinical trial*) or ((singl* or doubl* or treb* or tripl*) n1 (blind* or mask*)) or placebo* or (randomly allocated) or (allocated n2 random*)	87,580
S9	(DE "Clinical Trials") OR (DE "Placebo")	15,435
S8	(Mechanism* or process* or memor* or (cognitive function*) or ((negativ* or depressogenic*) n3 think*) or ruminat*)	1,497,463
S7	S5 OR S6	342,107
S6	(DE "Depression (Emotion)") OR (DE "Major Depression")	136,211
S5	depress*	342,107
S4	S1 OR S2 OR S3	228,613
S3	DE "Implosive Therapy"	1,017
S2	DE "Cognitive Therapy"	23,334
S1	(cognit* n5 behav*) or ((cognit* or behav* or conditioning or relaxation or desensiti*) n5 (therap* or psychotherap* or train* or retrain* or treat* or modif*)) or (implosive* n3 therap*)	228,613

Cochrane

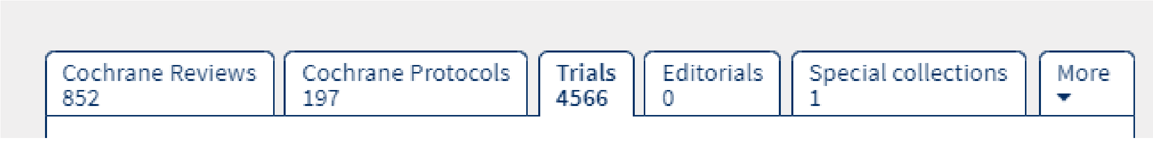

Search Name:
Date Run: 06/11/2018 10:10:46
Comment:

ID Search Hits
#1 ((cognit* near/5 behav*)):ti,ab,kw with Cochrane Library publication date from Nov null to present (Word variations have been searched) 14549
#2 ((cognit* or behav* or conditioning or relaxation or desensiti*) near/5 (therap* or psychotherap* or train* or retrain* or treat* or modif*)) 43009
#3 (implosive* near/3 therap*) 392
#4 MeSH descriptor: [Behavior Therapy] explode all trees 14454
#5 #1 or #2 or #3 or #4 47475
#6 MeSH descriptor: [Depression] explode all trees 9696
#7 MeSH descriptor: [Depressive Disorder] explode all trees 10298
#8 depress* 81488
#9 #6 or #7 or #8 81524
#10 (Mechanism* or process* or memor* or (cognitive function*) or ((negativ* or depressogenic*) near/3 think*) or ruminat*) 131389
#11 #5 and #9 and #10 5640

WHO Trials Registry

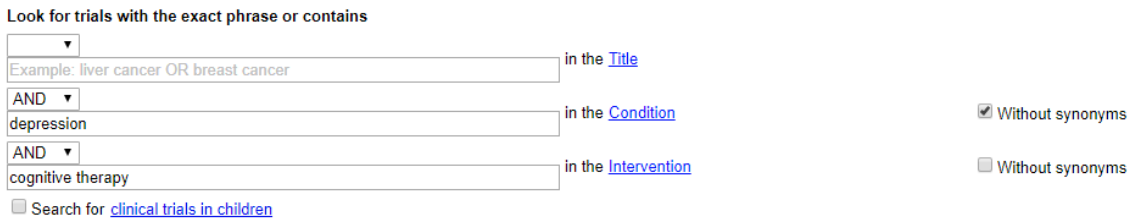

Supplement: Supplementary data [file bmjopen-2019-031110supp001.pdf]
