# Supplementary material for: Role of autobiographical memory in patient response to cognitive behavioural therapies for depression: protocol of an individual patient data meta-analysis
Source: BMJ Open. 2019 Jun 12;9(6):e031110. doi: 10.1136/bmjopen-2019-031110 (PMC6575822; doi:10.1136/bmjopen-2019-031110)
Supplement: Author's manuscript [file bmjopen-2019-031110.draft_revisions.pdf]

BMJ Open is committed to open peer review. As part of this commitment we make the peer review history of every article we publish publicly available.

When an article is published we post the peer reviewers' comments and the authors' responses online. We also post the versions of the paper that were used during peer review. These are the versions that the peer review comments apply to.

The versions of the paper that follow are the versions that were submitted during the peer review process. They are not the versions of record or the final published versions. They should not be cited or distributed as the published version of this manuscript.

BMJ Open is an open access journal and the full, final, typeset and author-corrected version of record of the manuscript is available on our site with no access controls, subscription charges or pay-per-view fees (http://bmjopen.bmj.com).

If you have any questions on BMJ Open's open peer review process please email info.bmjopen@bmj.com

BMJ Open

The role of autobiographical memory in patient response to cognitive behavioural therapies for depression: Protocol of an individual patient data meta-analysis

Journal:	*BMJ Open*
Manuscript ID	bmjopen-2019-031110
Article Type:	Protocol
Date Submitted by the Author:	16-Apr-2019

Complete List of Authors:	Hitchcock, Caitlin; University of Cambridge, MRC Cognition and Brain Sciences Unit; MRC Cognition and Brain Sciences Uni<br>Rudokaite, Judita; University of Cambridge, MRC Cognition and Brain Sciences Unit<br>Patel, Shivam; University of Cambridge, MRC Cognition and Brain Sciences Unit<br>Smith, Alicia; University of Cambridge, MRC Cognition and Brain Sciences Unit<br>Kuhn, Isla; University of Cambridge<br>Watkins, Edward<br>Dalgleish, Tim; University of Cambridge, MRC Cognition and Brain Sciences Unit

Keywords:	Depression & mood disorders < PSYCHIATRY, MENTAL HEALTH, Protocols & guidelines < HEALTH SERVICES ADMINISTRATION & MANAGEMENT

SCHOLARONE™
Manuscripts

The role of autobiographical memory in patient response to cognitive behavioural therapies for depression: Protocol of an individual patient data meta-analysis

Caitlin Hitchcock *
MRC Cognition and Brain Sciences Unit, University of Cambridge
Caitlin.hitchcock@mrc-cbu.cam.ac.uk

Judita Rudokaite
MRC Cognition and Brain Sciences Unit, University of Cambridge
juditarud@gmail.com

Shivam D Patel
MRC Cognition and Brain Sciences Unit, University of Cambridge
Shivam.patel@mrc-cbu.cam.ac.uk

Alicia Smith
MRC Cognition and Brain Sciences Unit, University of Cambridge
alicia.smith@student.manchester.ac.uk

Isla Kuhn
University of Cambridge
ilk21@cam.ac.uk

Ed Watkins
University of Exeter
E.R.Watkins@exeter.ac.uk

Tim Dalgleish
MRC Cognition and Brain Sciences Unit, University of Cambridge
Cambridgeshire and Peterborough NHS Foundation Trust
Tim.dalgleish@mrc-cbu.cam.ac.uk

* Corresponding author
15 Chaucer Road, Cambridge, CB2 7EF, United Kingdom

For peer review only

For peer review only - http://bmjopen.bmj.com/site/about/guidelines.xhtml

Abstract

Introduction. Cognitive behavioural therapies are one of the most effective treatments for major depression. However, approximately 50% of individuals do not adequately respond to intervention and of those who do remit from a depressive episode, approximately 50% will experience later relapse. Identification of patient-level factors which moderate treatment response may ultimately help to identify cognitive barriers that could be targeted to improve treatment efficacy. This individual patient data meta-analysis explores one such potential moderator - the ability to retrieve specific, detailed memories of the autobiographical past - as cognitive-based therapeutic techniques draw heavily on the ability to use specific autobiographical information to challenge the dysfunctional beliefs which drive depression.

Methods and analysis. We have formed a collaborative network which will contribute known datasets. This will be supplemented by datasets identified through literature searches in Medline, PsycInfo, Web of Science, the Cochrane Central Register of Controlled Trials, and World Health Organisation trials database. Inclusion criteria are delivery of a cognitive or cognitive-behavioural therapy for major depression, and measurement of autobiographical memory retrieval at pre-intervention. Primary outcomes are depressive symptoms and clinician-rated diagnostic status at post-intervention, along with autobiographical memory specificity at post-intervention. Secondary outcomes will consider each of these variables at follow-up. All analyses will be completed using random effects models employing restricted maximum likelihood estimation. Risk of bias in included studies will be measured using the Revised Cochrane Risk of Bias tool.

Ethics and Dissemination. The findings will be published in a peer-reviewed journal. Study results will contribute to better understanding of the role of autobiographical memory

For peer review only - http://bmjopen.bmj.com/site/about/guidelines.xhtml

in patient response to cognitive behavioural therapy, and may help to inform personalised medicine approaches to treatment of depression.

Registration: PROSPERO CRD42018109673

Strengths and limitations of this study

- This is the first individual patient data (IPD) meta-analysis of the role of autobiographical memory in patient response to cognitive behavioural therapy for major depression.
- This study may identify a patient-level moderator of response to cognitive behavioural therapies.
- Results will advance understanding of whether cognitive behavioural therapies improve this relapse risk factor.
- We anticipate that there may only be a small number of trials which have measured autobiographical memory, which may limit conclusions.

For peer review only

For peer review only - http://bmjopen.bmj.com/site/about/guidelines.xhtml

Introduction

Depression is the second leading cause of disability worldwide (Ferrari et al., 2013). The disorder costs the UK economy an estimated £7.5 billion per year, with this cost expected to increase (McCrone et al., 2008). Antidepressant medications (e.g., selective serotonin reuptake inhibitors; tricyclic antidepressants) are effective in treating depression (Fournier et al., 2010; Arroll et al., 2005), but only so long as they are taken (Altshuler et al., 2001; 2003). Despite efficacious psychological treatments such as interpersonal psychotherapy (de Mello et al., 2005; O'Hara et al., 2000), and cognitive behavioural therapies (CBTs: Butler et al., 2006, Hofmann et al., 2012), only 40-60% of NHS patients experience recovery after receiving psychological intervention for depression (Pybis et al., 2017). Of those who do respond, up to 50% will experience later relapse (Kessler et al., 1997). Our gold-standard psychological interventions are therefore producing suboptimal outcomes for a significant number of individuals.

Improving treatment success rates is therefore a key priority for social, economic, and health systems. Identification of patient-level factors which may interfere with the efficacy of psychological interventions and predispose an individual toward poorer treatment response may help to explain differential response rates. If we consider cognitive behavioural therapies, which are commonly administered in front-line NHS psychology services (e.g., Improving Access to Psychological Therapies services; Pybis et al., 2017), identification of cognitive barriers which may impede the ability to complete cognitive therapy tasks may advance our understanding of exactly why some individuals respond poorly, or not at all. Similarly, cognitive predictors of depression which are not shifted by intervention may indicate relapse risk factors (Gotlib & Joormann, 2010). Together, further understanding of patient-level factors that both impede treatment efficacy, and/or are not shifted by treatment

For peer review only - http://bmjopen.bmj.com/site/about/guidelines.xhtml

and go on to predict relapse may contribute to development of a personalised medicine approach (Simon & Perlis, 2010) for treatment of depression.

Autobiographical memory, our memory for personal life experiences, is one cognitive factor which plays an important role in the course of depression (Williams et al., 2007). In particular, the ability to retrieve specific, detailed memories of single incidents (e.g., *the occasion I met my partner*) predicts future depressive symptoms over and above current symptom levels (for a meta-analysis see Sumner et al., 2010). The ability to retrieve specific autobiographical memories not only independently predicts prognosis (e.g., by reducing the ability to solve problems or plan for the future; Jing et al., 2016, 2017), but may also impede the ability to complete CBT for depression.

A core aim of both traditional and later wave CBTs is to change the way that an individual relates to dysfunctional thoughts about the self and world (originally outlined by Beck, 1967). Key therapeutic techniques encourage the individual to identify links between thoughts and feelings, by recalling what they said to themselves, how that made them feel, and how that led them to act in a recent situation (e.g., *going to a work function*). For example, a core technique is to encourage the individual to not automatically accept thoughts as facts, but rather, to evaluate the accuracy of each thought (e.g., *no one wants to talk to me*) using specific pieces of evidence recalled from the individual's past (e.g., *last time I was at a work function I had a lovely conversation with a colleague*). The efficacy of such techniques is therefore likely to be influenced by an individual's ability to recall detailed, specific information about their autobiographical past. Treatment manuals instruct cognitive therapists to enrich detail and encourage specificity in patient responses while they are completing such tasks, however, a moderating role of memory specificity in the efficacy of CBTs has yet to be explored.

For peer review only

Similarly, it is possible that this reduction in autobiographical memory specificity may also be improved as a result of completing CBT. In continuously promoting a focus on specific information, and reducing the depressive tendency to overgeneralise information, CBT techniques may actively train an individual to be more specific. Indeed, increasing the specificity of thinking has been previously proposed as a key mechanism of effective CBT for depression (Watkins, 2009). There is some prior literature which suggests that autobiographical memory specificity may improve following a course of cognitive-based intervention, most notably CBT (e.g., McBride. Segal, Kennedy, & Gemar, 2007) and mindfulness-based cognitive therapy (MBCT; Williams, Teasdale, Segal, & Soulsby, 2000). In contrast, evidence that reduced memory specificity persists between depressive episodes (Sumner, Griffith, & Mineka, 2010) may indicate that this relapse risk factor is not commonly shifted by treatment. Determining the impact of CBTs on autobiographical memory specificity, relative to natural recovery, antidepressant medication and other active psychological interventions, will advance our understanding of how treatment impacts this relapse risk factor, and offer important implications for the adjunctive use of autobiographical memory-based interventions (for review see Barry, Lenaert, Hermans, Raes & Griffith, 2018; Hitchcock, Werner-Seidler, Blackwell, & Dalgleish, 2017) to enhance treatment outcomes.

The lack of prior investigation of the interaction between autobiographical memory specificity and CBTs has largely been due to a lack of statistical power to examine moderation effects in prior studies. Prior trials of CBTs which have included a measure of autobiographical memory have recruited small sample sizes (e.g., n=21 in the MBCT group in Williams et al., 2000), and thus been underpowered to explore these effects. Completion of individual patient data meta-analysis (IPD-MA) can overcome this issue. The IPD-MA approach involves synthesis of patient-level data across multiple studies, thereby offering greater statistical potential to explore individual characteristics and how these interact with

For peer review only - http://bmjopen.bmj.com/site/about/guidelines.xhtml

treatment effects, relative to aggregate meta-analysis which synthesises data at the study-level (Fisher, Copas, Tierney, & Parmar, 2011). We have therefore formed a collaborative network to allow us to complete an IPD-MA evaluating the nature of autobiographical memory specificity in patient response to cognitive behavioural treatment for depression.

Specifically, we will address the following questions:

1) First, does treatment for depression a) induce a change in autobiographical memory specificity? And b) induce a stronger change following CBTs relative to other interventions?
2) Second, does the specificity of autobiographical memory at baseline predict treatment response a) for all interventions (predictor effect); and b) is this effect different for CBTs relative to other interventions (moderation effect)?

Method

Study registration and management

This IPD-MA will be conducted in accordance with PRISMA guidelines (Moher et al., 2009), and is registered on PROSPERO, (CRD42018109673). Regular e-mail updates will be used to inform the collaborating network of our activities. Encrypted electronic data sharing clouds and e-mail will be used to exchange data and paperwork between researchers.

Criteria for included studies

Types of studies

Included studies will be both controlled and uncontrolled clinical trials. Studies may be randomised at patient level, cluster-randomised or non-randomised. For cross-over design studies, only data for the initial phase (i.e., pre cross-over) will be used. Articles must be published in English. Unpublished data will be actively sought; hence, non-peer reviewed studies will also be included. Sensitivity analyses will be completed to evaluate the impact of study type on our results.

For peer review only - http://bmjopen.bmj.com/site/about/guidelines.xhtml

For peer review only

Participants

Studies must have recruited participants aged 18 years or older. Participants must have a diagnosis of Major Depressive Disorder (MDD) according to the Diagnostic and Statistical Manual of Mental Disorders (any edition) or International Classification of Diseases (any edition) criteria, assessed via a structured clinical interview (e.g., Structured Clinical Interview for DSM Disorders; Mini International Neuropsychiatric Interview). As depression-related deficits in autobiographical memory are evident into remission (Sumner et al., 2010) and National Institute of Health and Care Excellence (NICE) guidelines recommend that CBT interventions are used to reduce depressive relapse (e.g., MBCT; NICE, 2009), participants may either be experiencing a current major depressive episode, or remitted from MDD.

Autobiographical memory

Studies must include an objective measure of the specificity of autobiographical memory. This may be indexed using a cued-recalled task (e.g., the Autobiographical Memory Task; Williams & Broadbent, 1986), an interview (e.g., Autobiographical Interview; Levine, Svoboda, Hay, Winocur, & Moscovitch, 2002), or sentence completion procedure (e.g., Raes et al., 2007) but responses must be scored for the number of specific, single incident memories retrieved.

Intervention

We will include studies evaluating any psychological therapy for depression which is cognitively-based as per the Beck model (e.g., cognitive therapy, cognitive behavioural therapy, mindfulness-based cognitive therapy). This will be determined by two independent reviewers using the author's intervention description. Interventions may be self-guided or therapist-guided, and delivered in-person or online, and in individual or group-based format. Excluded interventions will be those which directly aim to improve a defined cognitive

For peer review only - http://bmjopen.bmj.com/site/about/guidelines.xhtml

process or bias (e.g., cognitive/attention/interpretative bias modification, autobiographical memory-based intervention, concreteness training).

Comparison condition

Studies may include no comparison condition (i.e., uncontrolled trials) or may include any type of comparison condition. Comparison conditions are anticipated to be waitlist, any psychosocial intervention (e.g., psychoeducation, counselling), or antidepressant medication. Again, condition type will be determined by two independent reviewers based on the author's intervention description. CBTs will be compared a) against waitlist control (to index effects against natural recovery), b) against all other interventions, c) against other psychological interventions alone and d) against antidepressant medication alone.

Co-primary outcomes

For Question 1, the primary outcome will be autobiographical memory specificity at post-intervention. This will be operationalised as the proportion of specific, single incident memories reported by the participant during an objective measure of autobiographical memory. Specificity of memories will be rated by researchers (not participants). Proportions (number specific/number of trials) will be calculated for any studies reporting the number of specific memories.

For Question 2, the co-primary outcomes will be treatment response at post-intervention, indexed as a) a score on a continuous measure of depressive symptoms and b) diagnostic status, as rated using a standardised clinician-administered interview. In situations where more than one continuous measure of depression is administered, the Beck Depression Inventory II (BDI-II) will be preferred in analysis as this has been specifically devised to measure changes following CBT interventions. Any studies reporting Beck Depression Inventory I scores will be converted to BDI-II scores using the method outlined in the BDI-II manual (Beck, Steer, & Brown, 1996).

For peer review only - http://bmjopen.bmj.com/site/about/guidelines.xhtml

Secondary outcomes

As it is highly desirable for an intervention to produce stable, lasting change, follow-up assessments will be considered as secondary outcomes. The secondary outcomes for Question 1 will be autobiographical memory specificity (as per defined above) at follow-up assessments. The secondary outcome for Question 2 will be depressive symptoms and diagnostic status (as per defined above) at follow-up assessments.

Follow-up lengths to be included are assessments between one month and two years following the completion of therapy. During analysis, studies including a follow-up assessment between one and three months post-intervention will be grouped to form a short-term follow-up, and any later assessment points will be grouped per six month period (i.e., 6 months, 12 months). This will result in analysis of follow-up outcomes in the short-term (1-3 months), and at 6, 12, 18, and 24 months post-intervention.

Moderator

For Question 2, the moderating variable will be the proportion of specific, single incident memories reported by the participant during an objective measure of autobiographical memory at baseline.

Search methods for identification of studies

Collaborative network. We have formed a collaborative network of experts in the role of autobiographical memory in depression. Through our own work and knowledge of the field, we have initially identified and obtained access to four individual datasets which provide the data necessary to analyse our research questions. Using this network to identify other potential datasets for inclusion, we will email the autobiographical memory and psychopathology special interest group, and the collaborators of each member of our network. We will also make use of social media such as Twitter to raise awareness of the IPD-MA. This mode of identification is important, as we anticipate that many of the trials

For peer review only

For peer review only - http://bmjopen.bmj.com/site/about/guidelines.xhtml

which have included an autobiographical memory measure may not list the measure in the method section of the published paper. Indeed, this is the case for the majority of the datasets the collaborative network has already identified.

Electronic searches. As we anticipate that many of the trials which have included an autobiographical memory measure may not list this measure in the published paper, a multi-stage search strategy will be completed in order to identify all potential datasets.

First, searches will be completed in PsycINFO, Medline, Web of Science, Cochrane database, and WHO trials database, using the string: cognitive therapy[1] AND depression AND trial AND any flag words (with OR inserted between every flag word*) we have identified as markers of papers which may potentially include an autobiographical memory measure. These flag words were identified by evaluating common terminology in the abstracts of the four datasets identified through the collaboration network. These terms are: 'mechanism,' 'process', 'memory', 'cognitive function', 'negative thinking', 'rumination', 'depressogenic thinking', 'autobiographical memory'. Searches will be restricted to papers published in or after 1986 as this was the year that the seminal paper on autobiographical memory specificity in depression was published (Williams & Broadbent, 1986). The full electronic search strategy is available in the Supplementary Materials.

Search results will be imported into Rayyan (a web-based tool for managing systematic reviews) by an administrator (Kuhn) and duplicates will be removed. This will allow for blind screening by two independent reviewers (Rudokaite and Patel). Papers deemed ineligible from title and abstract by both reviewers will be set aside. Full text of the remaining papers will be examined independently by the reviewers to determine eligibility.

[1] We use the term 'cognitive therapy' although we will also include cognitive behavioural therapies (CBTs) as use of the term 'cognitive behavioural therapy' may miss studies which administer cognitive therapy. Conversely, searches using the term 'cognitive therapy' did identify evaluations of CBTs.

For peer review only - http://bmjopen.bmj.com/site/about/guidelines.xhtml

Any discrepancy in inclusion will be resolved via discussion with Hitchcock, or if necessary, by contacting the study authors for clarification.

Next, a secondary screening will be completed in order to identify studies which may have measured autobiographical memory but not reported on it in the published paper. Through searches in Medline, PsycINFO and Web of Science using the terms autobiographical memory AND depression, we will produce a list of authors who have ever published on autobiographical memory and depression. This list will then be cross-referenced with the results of the primary database search. For studies with an author who has previously published on autobiographical memory, corresponding authors will then be emailed to enquire about unpublished autobiographical memory data.

Data collection

Corresponding authors of eligible studies will be emailed to request data. A reminder email will be sent every two weeks. If an author does not respond after 1 month, another author of the study will be contacted. A second attempt to contact this author will follow and so forth until a maximum of three authors are contacted. Study data will be considered unavailable if no study authors have responded to multiple contact attempts, or if authors indicate that they no longer have access to the data.

The authors will be able to supply data via email or an encrypted online data sharing service operated by the University of Cambridge. A single author will be identified for each study to whom all queries about the data collection processes and transformation of individual variables will be addressed. When researchers are cleaning a specific data set they may communicate with the original investigators via telephone discussions or by email.

Data extraction, quality checks, and storage

In accordance with Cochrane recommendations, both published and unpublished measures will be collected for all studies. Individual patient data for specified variables

For peer review only

(presented in Table 1) will be extracted into a single dataset. For all outcomes, unimputed and untransformed data will be preferred. Spot checks will be completed to ensure data quality. The pattern of treatment allocation for each included study will be checked to ensure that blinding, randomization and allocation sequence appear appropriate, in accordance with guidelines recommended by Tierney et al. (2015). Data will be stored in password protected files on an encrypted University of Cambridge server, and will not include any personally identifiable information.

Table 1. Individual patient data to be extracted from included studies

Trial-level data	Patient characteristics	Outcomes
Trial identifier	Anonymised participant identifier	Post-treatment self-reported depressive symptoms
Country of completion	Age	Follow-up self-reported depressive symptoms
Type of CBT	Gender	Diagnostic status at post-treatment
Type of comparison group/s	Education history	Diagnostic status at follow-up
Format of treatment (individual, group)	Ethnicity	Post-treatment score for proportion of specific memories
Length of treatment in weeks	Concurrent treatment use (e.g., medication)	Post-treatment score for proportion of non-specific memories
Information about risk of bias	Pre-treatment self-reported depressive symptoms	Follow-up score for proportion of specific memories
Trial design		
	Pre-treatment score for number of specific memories	Follow-up score for proportion of non-specific memories
	Pre-treatment score for number of non-specific memories	
	Any potential cognitive covariates (e.g., rumination, working memory, IQ, executive function)	
	Reasons for missing data	

For peer review only

For peer review only - http://bmjopen.bmj.com/site/about/guidelines.xhtml

Risk of bias

For individual studies, risk of bias will be evaluated using the Revised Cochrane Risk of Bias Tool (Higgins et al., 2016) to access study quality and risk of bias due to the randomisation process, deviations from intended interventions, missing outcome data, measurement of the outcome, selection of the reported result and other biases such as conflicts of interest. Using the tool, each study will be rated as of high-, low-, or unclear risk. In order to reduce attrition bias, data will be checked against trial CONSORT diagrams to ensure that data from all randomised participants are included.

Strategy for data synthesis

Intent-to-treat analysis will be completed using data imputed at within-study level. Between-study heterogeneity will be indexed using the tau statistic. As there are now multiple IPD-MAs establishing the effect of CBTs on depressive outcomes (e.g., Furukawa et al., 2018; Weitz, Hollon, Twisk, & et al., 2015), we will proceed directly to evaluating moderation effects.

All analyses will be completed using random effects models employing restricted maximum likelihood estimation. However, if there is considerable heterogeneity in the quality of studies (indexed by the Cochrane Risk of Bias tool) sensitivity analysis will be completed using fixed effects models such that poorer quality studies will not receive equal weighting. If IPD are available for more than seven studies, prediction intervals will be used (Partlett & Riley, 2017). If we obtain sufficient power, subsets of studies will be analysed to explore whether the below effects vary based on trauma-history (as trauma exposure may reduce memory specificity; Moore & Zoellner, 2007), depressive severity, and type of therapy (e.g., MBCT, CBT).

Question 1. The effect of intervention on autobiographical memory specificity will be analysed using an ANCOVA random treatment effect one-stage model with random intercept

For peer review only - http://bmjopen.bmj.com/site/about/guidelines.xhtml

and memory specificity baseline adjustment with different residual variance per study. Sensitivity analysis will be completed using stratified intercepts.

Question 2.

To determine whether autobiographical memory specificity moderates treatment response, a two-stage model predicting post-treatment depressive symptoms with baseline symptom adjustment is planned to allow synthesis of datasets which used different symptom measures (e.g., BDI-II and Hamilton Depression Rating Scale [HDRS]). The interaction term (baseline score x treatment type) will be estimated per trial, then pooled in a random effects meta-analysis. Sensitivity analysis will next be completed using separate one-stage models for different depressive symptom measures. Patient-level covariates will be centred to separate within-trial and across-trial effects.

Effects on diagnostic status will be analysed using a one-stage logistic model with random intercept to account for correlation between the interaction estimate and other parameter estimates. Again, patient-level covariates will be centred to separate within-trial and across-trial effects. Sensitivity analysis will be completed using stratified intercepts. If the one-stage model fails to converge, a two-stage model will be completed.

Ethics and dissemination

Ethics approval was obtained from the Cambridge Psychology Research Ethics Committee (Identifier PRE.2017.100). Contributing studies will be asked to provide evidence of local ethical committee approval and to demonstrate that informed consent has been given to share de-identifed data. Contributing studies will also be required to remove patient identifiers before providing their data. This includes date of birth, which will be converted to age at time of assessment. A data sharing agreement will be signed between the authors of included studies and the IPD-MA research team. The resulting IPD-MA dataset will be

For peer review only - http://bmjopen.bmj.com/site/about/guidelines.xhtml

shared through the MRC Cognition and Brain Sciences Unit Data Repository (accessed via www.mrc-cbu.cam.ac.uk/publications/opendata/) in accordance with open-science practices.

Patient and Public Involvement: Members of the PPI group formed by individuals with lived experience of depression discussed the project and the role of specificity in CBTs, and will contribute to dissemination of results.

Authors' contributions: CH, TD, & EW designed the project. JR, SDP, AS, & IK contributed to study methods. All authors contributed to writing the paper.

Funding statement: This work was funded by an ESRC grant (Ref: ES/R010781/1) awarded to the first author.

Competing interests statement. None.

Word count: 3831

For peer review only

Altshuler, L., Kiriakos, L., Calcagno, J., Goodman, R., Gitlin, M., Frye, M., & Mintz, J. (2001). The impact of antidepressant discontinuation versus antidepressant continuation on 1-year risk for relapse of bipolar depression: a retrospective chart review. *The Journal of Clinical Psychiatry.*

Altshuler, L., Suppes, T., Black, D., Nolen, W. A., Keck Jr, P. E., Frye, M. A., ... & Leverich, G. (2003). Impact of antidepressant discontinuation after acute bipolar depression remission on rates of depressive relapse at 1-year follow-up. *American Journal of Psychiatry*, *160*(7), 1252-1262.

Arroll, B., Macgillivray, S., Ogston, S., Reid, I., Sullivan, F., Williams, B., & Crombie, I. (2005). Efficacy and tolerability of tricyclic antidepressants and SSRIs compared with placebo for treatment of depression in primary care: a meta-analysis. *The Annals of Family Medicine*, *3*(5), 449-456.

Barry, T. J., Lenaert, B., Hermans, D., Raes, F., & Griffith, J. W. (2018). Meta-Analysis of the Association Between Autobiographical Memory Specificity and Exposure to Trauma. *Journal of Traumatic Stress, 31*(1), 35-46. doi:doi:10.1002/jts.22263.

Beck, A. T. (1967). *Depression: Clinical, Experimental, and Theoretical Aspects*: Hoeber Medical Division, Harper & Row.

Beck, A.T., Steer, R.A., Ball, R., and Ranieri, W.F. (1996). Comparison of Beck Depression Inventories-IA and-II in Psychiatric Outpatients. *Journal of Personality Assessment*, *67*(3), 588-597.

Butler, A. C., Chapman, J. E., Forman, E. M., & Beck, A. T. (2006). The empirical status of cognitive-behavioral therapy: a review of meta-analyses. *Clinical Psychology Review*, *26*(1), 17-31.

For peer review only - http://bmjopen.bmj.com/site/about/guidelines.xhtml

de Mello, M. F., de Jesus Mari, J., Bacaltchuk, J., Verdeli, H., & Neugebauer, R. (2005). A systematic review of research findings on the efficacy of interpersonal therapy for depressive disorders. *European Archives of Psychiatry and Clinical Neuroscience*, *255*(2), 75-82.

Ferrari, A. J., Charlson, F. J., Norman, R. E., Patten, S. B., Freedman, G., Murray, C. J., ... & Whiteford, H. A. (2013). Burden of depressive disorders by country, sex, age, and year: findings from the global burden of disease study 2010. *PLoS Medicine*, *10*(11), e1001547.

Fisher, D., Copas, A., Tierney, J., & Parmar, M. (2011). A critical review of methods for the assessment of patient-level interactions in individual participant data meta-analysis of randomized trials, and guidance for practitioners. *Journal of Clinical Epidemiology, 64*(9), 949-967. doi:10.1016/j.jclinepi.2010.11.016.

Fournier, J. C., DeRubeis, R. J., Hollon, S. D., Dimidjian, S., Amsterdam, J. D., Shelton, R. C., & Fawcett, J. (2010). Antidepressant drug effects and depression severity: a patient-level meta-analysis. *JAMA*, *303*(1), 47-53.

Furukawa, T. A., Efthimiou, O., Weitz, E. S., Cipriani, A., Keller, M. B., Kocsis, J. H., . . . Schramm, E. (2018). Cognitive-Behavioral Analysis System of Psychotherapy, Drug, or Their Combination for Persistent Depressive Disorder: Personalizing the Treatment Choice Using Individual Participant Data Network Metaregression. *Psychotherapy and Psychosomatics, 87*(3), 140-153. doi:10.1159/000489227.

Gotlib, I. H., & Joormann, J. (2010). Cognition and Depression: Current Status and Future Directions. *Annual Review of Clinical Psychology, 6*, 285-312. doi:10.1146/annurev.clinpsy.121208.131305.

Higgins, J.P.T., Sterne, J.A.C., Savović, J., Page, M.J., Hróbjartsson, A., Boutron, I., Reeves, B. & Eldridge, S. A revised tool for assessing risk of bias in randomized trials In:

For peer review only - http://bmjopen.bmj.com/site/about/guidelines.xhtml

Chandler, J., McKenzie, J., Boutron, I., Welch, V. (editors). *Cochrane Methods. Cochrane Database of Systematic Reviews* 2016, Issue 10 (Suppl 1). dx.doi.org/10.1002/14651858.CD201601.

Hitchcock, C., Werner-Seidler, A., Blackwell, S.E., and Dalgleish, T. (2017). Autobiographical episodic memory-based training for the treatment of mood, anxiety and stress-related disorders: A systematic review and meta-analysis. *Clinical Psychology Review*, *52*, 92-107. doi: 10.1016/j.cpr.2016.12.003

Hofmann, S. G., Asnaani, A., Vonk, I. J., Sawyer, A. T., & Fang, A. (2012). The efficacy of cognitive behavioral therapy: A review of meta-analyses. *Cognitive Therapy and Research*, *36*(5), 427-440.

Jing, H.G., Madore, K.P., and Schacter, D.L. (2016). Worrying About the Future: An Episodic Specificity Induction Impacts Problem Solving, Reappraisal, and Well-Being. *Journal of Experimental Psychology: General*, *145*(4), 402-418.

Jing, H.G., Madore, K.P., and Schacter, D.L. (2017). Preparing for what might happen: An episodic specificity induction impacts the generation of alternative future events. *Cognition*, *169*, 118-128.

Kessler. R.C., Zhao, S., Blazer, D.G., and Swartz, M. (1997). Prevalence, correlates, and course of minor depression and major depression in the National Comorbidity Survey. *Journal of Affective Disorders*, *45*(1-2), 19-30

Levine, B., Svoboda, E., Hay, J. F., Winocur, G., & Moscovitch, M. (2002). Aging and autobiographical memory: dissociating episodic from semantic retrieval. *Psychology and Aging, 17*(4), 677. doi:10.1037//0882-7974.17.4.677.

McBride, C., Segal, Z., Kennedy, S., and Gemar, M. (2007). Changes in autobiographical memory specificity following cognitive behavior therapy and pharmacotherapy for major depression. *Psychopathology*, *40*(3), 147-152. doi: 10.1159/000100003.

For peer review only

For peer review only - http://bmjopen.bmj.com/site/about/guidelines.xhtml

McCrone, P., et al. (2008). Paying the price. The cost of mental health care in England to 2026. *London: King's Fund*.

Moher D., Liberati, A., Tetzlaff J., Altman, D.G, and The PRISMA Group. (2009). Preferred Reporting Items for Systematic Reviews and Meta-Analyses: The PRISMA Statement. *PLOS Medicine*, *6*(7), e1000097, https://doi.org/10.1371/journal.pmed.1000097

Moore, S. A., & Zoellner, L. A. (2007). Overgeneral autobiographical memory and traumatic events: an evaluative review. *Psychological bulletin*, *133*(3), 419–437. doi:10.1037/0033-2909.133.3.419

O'Hara, M. W., Stuart, S., Gorman, L. L., & Wenzel, A. (2000). Efficacy of interpersonal psychotherapy for postpartum depression. *Archives of General Psychiatry*, *57*(11), 1039-1045.

Partlett, C., & Riley, R. D. (2017). Random effects meta-analysis: Coverage performance of 95% confidence and prediction intervals following REML estimation. *Statistics in Medicine, 36*(2), 301-317. doi:doi:10.1002/sim.7140.

Pybis, J., et al. (2017). The comparative effectiveness and efficiency of cognitive behaviour therapy and generic counselling in the treatment of depression: evidence from the 2nd UK National Audit of psychological therapies. *BMC Psychiatry*, *17*(1), 215-238.

Raes, F., Hermans, D., Williams, J. M. G., & Eelen, P. (2007). A sentence completion procedure as an alternative to the Autobiographical Memory Test for assessing overgeneral memory in non-clinical populations. *Memory*, *15*(5), 495-507.

Simon, G. E., & Perlis, R. H. (2010). PERSONALIZED MEDICINE FOR DEPRESSION: CAN WE MATCH PATIENTS WITH TREATMENTS? *The American Journal of Psychiatry*, *167*(12), 1445–1455. http://doi.org/10.1176/appi.ajp.2010.09111680.

For peer review only - http://bmjopen.bmj.com/site/about/guidelines.xhtml

Sumner, J. A., Griffith, J. W., & Mineka, S. (2010). Overgeneral autobiographical memory as a predictor of the course of depression: A meta-analysis. *Behaviour Research and Therapy, 48*(7), 614-625. doi:http://dx.doi.org/10.1016/j.brat.2010.03.013.

Tierney, J.F., Vale, C., Riley, R., Tudur Smith, C., Stewart, L., Clarke, M., and Rovers, M. (2015). Individual Participant Data (IPD) Meta-analyses of Randomised Controlled Trials: Guidance on Their Use. *PLOS Medicine*, *12*(7), e1001855. https://doi.org/10.1371/journal.pmed.1001855.

Watkins, E. R. (2009). Depressive rumination: investigating mechanisms to improve cognitive behavioural treatments. *Cognitive Behaviour Therapy*, *38*(S1), 8-14.

Weitz, E. S., Hollon, S. D., Twisk, J., & et al. (2015). Baseline depression severity as moderator of depression outcomes between cognitive behavioral therapy vs pharmacotherapy: An individual patient data meta-analysis. *JAMA Psychiatry, 72*(11), 1102-1109. doi:10.1001/jamapsychiatry.2015.1516.

Williams, J. M., Barnhofer, T., Crane, C., Herman, D., Raes, F., Watkins, E., & Dalgleish, T. (2007). Autobiographical memory specificity and emotional disorder. *Psychological bulletin*, *133*(1), 122–148. doi:10.1037/0033-2909.133.1.122

Williams, J. M., & Broadbent, K. (1986). Autobiographical memory in suicide attempters. *Journal of Abnormal Psychology, 95*(2), 5. doi:10.1037/0021-843X.95.2.144

Williams J. M. G., Teasdale J. D., Segal Z. V., & Soulsby J. (2000). Mindfulness-based cognitive therapy reduces overgeneral autobiographical memory in formerly depressed patients. *Journal of Abnormal Psychology*, *109*(1), 150–155

For peer review only - http://bmjopen.bmj.com/site/about/guidelines.xhtml

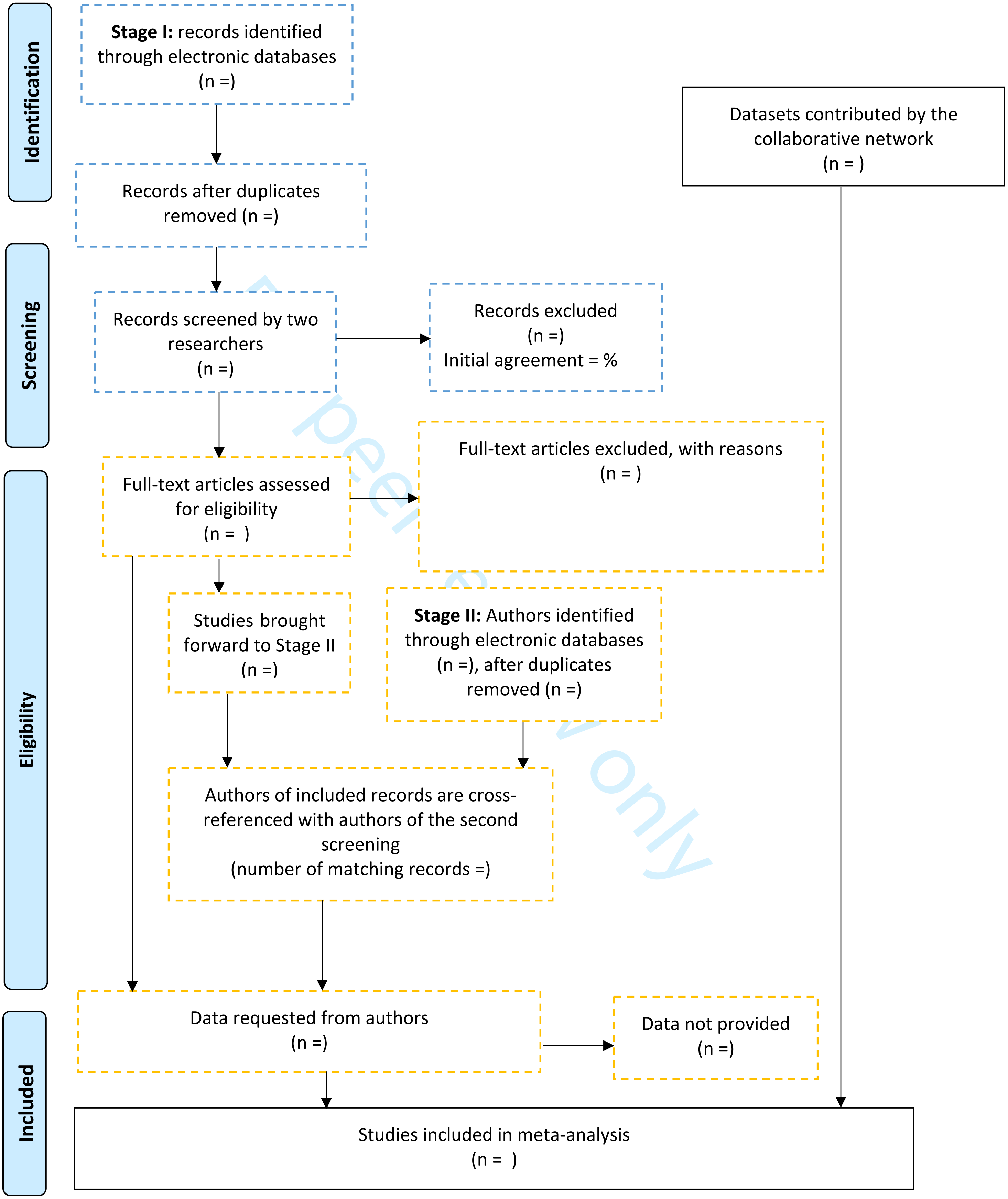
Identification
Screening
Eligibility
Included
Stage I: records identified through electronic databases (n =)
Records after duplicates removed (n =)
Datasets contributed by the collaborative network (n =)
Records screened by two researchers (n =)
Records excluded (n =) Initial agreement = %
Full-text articles excluded, with reasons (n =)
Full-text articles assessed for eligibility (n =)
Studies brought forward to Stage II (n =)
Stage II: Authors identified through electronic databases (n =), after duplicates removed (n =)
Authors of included records are cross-referenced with authors of the second screening (number of matching records =)
Data requested from authors (n =)
Data not provided (n =)
Studies included in meta-analysis (n =)

Overview – Stage 1: CBT & Depression – RCT - keywords

	Hits
Medline	1746
Web of Science	3994
PsycINFO	1189
Cochrane – Trials Database	4566
WHO	149
**Total**	**11637**
**Deduplication**	**8719**

No limits were applied in terms of pu

Medline

Database: Ovid MEDLINE(R) and Epub Ahead of Print, In-Process & Other Non-Indexed Citations, Daily and Versions(R) <1946 to November 5, 2018>

Search Strategy:

----

1 exp behavior therapy/ or (cognit* adj5 behav*).mp. or ((cognit* or behav* or conditioning or relaxation or desensiti*) adj5 (therap* or psychotherap* or train* or retrain* or treat* or modif*)).mp. or (implosive* adj3 therap*).mp.

2 exp depression/ or exp depressive disorder/ or depress*.mp.

3 (Randomized Controlled Trials as Topic/ or randomized controlled trial/ or Random Allocation/ or Double Blind Method/ or Single Blind Method/ or clinical trial/ or clinical trial, phase i.pt. or clinical trial, phase ii.pt. or clinical trial, phase iii.pt. or clinical trial, phase iv.pt. or controlled clinical trial.pt. or randomized controlled trial.pt. or multicenter study.pt. or clinical trial.pt. or exp Clinical Trials as topic/ or (clinical adj trial$).tw. or ((singl$ or doubl$ or treb$ or tripl$) adj (blind$3 or mask$3)).tw. or PLACEBOS/ or placebo$.tw. or randomly allocated.tw. or (allocated adj2 random$).tw.) not (case report.tw. or letter/ or historical article/)

4 (Mechanism* or process* or memor* or (cognitive adj function*) or ((negativ* or depressogenic*) adj3 think*) or ruminat*).mp.

5 1 and 2 and 3 and 4

Web of Science

5 #4 AND #3 AND #2 AND #1

For peer review only

Indexes=SCI-EXPANDED, SSCI, A&HCI, CPCI-S, CPCI-SSH, BKCI-S, BKCI-SSH, ESCI, CCR-EXPANDED, IC Timespan=All years

4 **TOPIC:** ((Mechanism* or process* or memor* or (cognitive near/1 function*) or ((negativ* or depressogenic*) near/3 think*) or ruminat*))
Indexes=SCI-EXPANDED, SSCI, A&HCI, CPCI-S, CPCI-SSH, BKCI-S, BKCI-SSH, ESCI, CCR-EXPANDED, IC Timespan=All years

3 **TOPIC:** (trial* or ((singl* or doubl* or trebl* or tripl*) near/1 (blind* or mask*)) or random*)
Indexes=SCI-EXPANDED, SSCI, A&HCI, CPCI-S, CPCI-SSH, BKCI-S, BKCI-SSH, ESCI, CCR-EXPANDED, IC Timespan=All years

2 **TOPIC:** (depress*)
Indexes=SCI-EXPANDED, SSCI, A&HCI, CPCI-S, CPCI-SSH, BKCI-S, BKCI-SSH, ESCI, CCR-EXPANDED, IC Timespan=All years

1 **TOPIC:** ((cognit* near/5 behav*) or ((cognit* or behav* or conditioning or relaxation or desensiti*) near/5 (therap* or psychotherap* or train* or retrain* or treat* or modif*)) or (implosive* near/3 therap*))
Indexes=SCI-EXPANDED, SSCI, A&HCI, CPCI-S, CPCI-SSH, BKCI-S, BKCI-SSH, ESCI, CCR-EXPANDED, IC Timespan=All years

PsycINO

#	Query	Results
S12	S4 AND S7 AND S8 AND S11	1,189
S11	S9 OR S10	87,580
S10	(clinical trial*) or ((singl* or doubl* or treb* or tripl*) n1 (blind* or mask*)) or placebo* or (randomly allocated) or (allocated n2 random*)	87,580
S9	(DE "Clinical Trials") OR (DE "Placebo")	15,435
S8	(Mechanism* or process* or memor* or (cognitive function*) or ((negativ* or depressogenic*) n3 think*) or ruminat*)	1,497,463
S7	S5 OR S6	342,107
S6	(DE "Depression (Emotion)") OR (DE "Major Depression")	136,211
S5	depress*	342,107
S4	S1 OR S2 OR S3	228,613
S3	DE "Implosive Therapy"	1,017
S2	DE "Cognitive Therapy"	23,334
S1	(cognit* n5 behav*) or ((cognit* or behav* or conditioning or relaxation or desensiti*) n5 (therap* or psychotherap* or train* or retrain* or treat* or modif*)) or (implosive* n3 therap*)	228,613

For peer review only - http://bmjopen.bmj.com/site/about/guidelines.xhtml

Cochrane

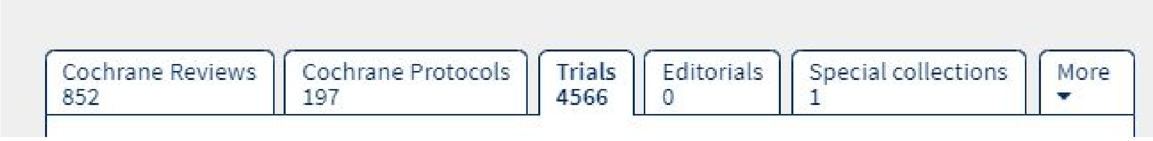

Search Name:
Date Run: 06/11/2018 10:10:46
Comment:

ID Search Hits
#1 ((cognit* near/5 behav*)):ti,ab,kw with Cochrane Library publication date from Nov null to present (Word variations have been searched) 14549
#2 ((cognit* or behav* or conditioning or relaxation or desensiti*) near/5 (therap* or psychotherap* or train* or retrain* or treat* or modif*)) 43009
#3 (implosive* near/3 therap*) 392
#4 MeSH descriptor: [Behavior Therapy] explode all trees 14454
#5 #1 or #2 or #3 or #4 47475
#6 MeSH descriptor: [Depression] explode all trees 9696
#7 MeSH descriptor: [Depressive Disorder] explode all trees 10298
#8 depress* 81488
#9 #6 or #7 or #8 81524
#10 (Mechanism* or process* or memor* or (cognitive function*) or ((negativ* or depressogenic*) near/3 think*) or ruminat*) 131389
#11 #5 and #9 and #10 5640

WHO Trials Registry

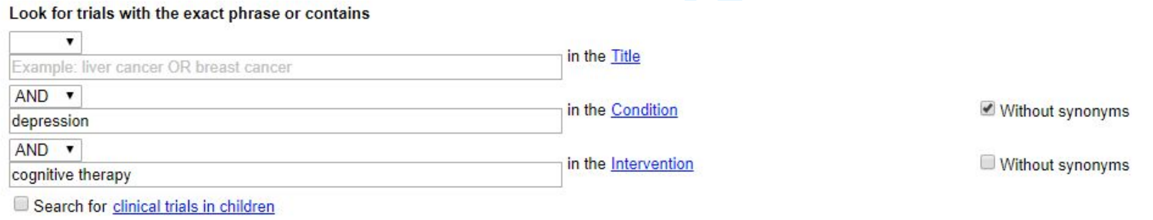

For peer review only - http://bmjopen.bmj.com/site/about/guidelines.xhtml

PRISMA-P (Preferred Reporting Items for Systematic review and Meta-Analysis Protocols) 2015 checklist: recommended items to address in a systematic review protocol*

Section and topic	Item No	Checklist item	(Page No.#)
**ADMINISTRATIVE INFORMATION**			
Title:			
Identification	1a	Identify the report as a protocol of a systematic review	1
Update	1b	If the protocol is for an update of a previous systematic review, identify as such	NA
Registration	2	If registered, provide the name of the registry (such as PROSPERO) and registration number	3
Authors:			
Contact	3a	Provide name, institutional affiliation, e-mail address of all protocol authors; provide physical mailing address of corresponding author	1
Contributions	3b	Describe contributions of protocol authors and identify the guarantor of the review	16
Amendments	4	If the protocol represents an amendment of a previously completed or published protocol, identify as such and list changes; otherwise, state plan for documenting important protocol amendments	NA
Support:			
Sources	5a	Indicate sources of financial or other support for the review	16
Sponsor	5b	Provide name for the review funder and/or sponsor	
Role of sponsor or funder	5c	Describe roles of funder(s), sponsor(s), and/or institution(s), if any, in developing the protocol	
**INTRODUCTION**			
Rationale	6	Describe the rationale for the review in the context of what is already known	4
Objectives	7	Provide an explicit statement of the question(s) the review will address with reference to participants, interventions, comparators, and outcomes (PICO)	7
**METHODS**			
Eligibility criteria	8	Specify the study characteristics (such as PICO, study design, setting, time frame) and report characteristics (such as years considered, language, publication status) to be used as criteria for eligibility for the review	7-10
Information sources	9	Describe all intended information sources (such as electronic databases, contact with study authors, trial registers or other grey literature sources) with planned dates of coverage	10-12
Search strategy	10	Present draft of search strategy to be used for at least one electronic database, including planned limits, such that it could be repeated	11 and supplementary

For peer review only

			materials
Study records:			
Data management	11a	Describe the mechanism(s) that will be used to manage records and data throughout the review	12
Selection process	11b	State the process that will be used for selecting studies (such as two independent reviewers) through each phase of the review (that is, screening, eligibility and inclusion in meta-analysis)	11
Data collection process	11c	Describe planned method of extracting data from reports (such as piloting forms, done independently, in duplicate), any processes for obtaining and confirming data from investigators	12
Data items	12	List and define all variables for which data will be sought (such as PICO items, funding sources), any pre-planned data assumptions and simplifications	Table 1
Outcomes and prioritization	13	List and define all outcomes for which data will be sought, including prioritization of main and additional outcomes, with rationale	9
Risk of bias in individual studies	14	Describe anticipated methods for assessing risk of bias of individual studies, including whether this will be done at the outcome or study level, or both; state how this information will be used in data synthesis	14
Data synthesis	15a	Describe criteria under which study data will be quantitatively synthesised	14
	15b	If data are appropriate for quantitative synthesis, describe planned summary measures, methods of handling data and methods of combining data from studies, including any planned exploration of consistency (such as I^2, Kendall's τ)	
	15c	Describe any proposed additional analyses (such as sensitivity or subgroup analyses, meta-regression)	
	15d	If quantitative synthesis is not appropriate, describe the type of summary planned	
Meta-bias(es)	16	Specify any planned assessment of meta-bias(es) (such as publication bias across studies, selective reporting within studies)	14
Confidence in cumulative evidence	17	Describe how the strength of the body of evidence will be assessed (such as GRADE)	14

*** It is strongly recommended that this checklist be read in conjunction with the PRISMA-P Explanation and Elaboration (cite when available) for important clarification on the items. Amendments to a review protocol should be tracked and dated. The copyright for PRISMA-P (including checklist) is held by the PRISMA-P Group and is distributed under a Creative Commons Attribution Licence 4.0.**

From: Shamseer L, Moher D, Clarke M, Ghersi D, Liberati A, Petticrew M, Shekelle P, Stewart L, PRISMA-P Group. Preferred reporting items for systematic review and meta-analysis protocols (PRISMA-P) 2015: elaboration and explanation. BMJ. 2015 Jan 2;349(jan02 1):g7647.

For peer review only

BMJ Open

The role of autobiographical memory in patient response to cognitive behavioural therapies for depression: Protocol of an individual patient data meta-analysis

Journal:	*BMJ Open*
Manuscript ID	bmjopen-2019-031110.R1
Article Type:	Protocol
Date Submitted by the Author:	26-Apr-2019
Complete List of Authors:	Hitchcock, Caitlin; University of Cambridge, MRC Cognition and Brain Sciences Unit; MRC Cognition and Brain Sciences Uni<br>Rudokaite, Judita; University of Cambridge, MRC Cognition and Brain Sciences Unit<br>Patel, Shivam; University of Cambridge, MRC Cognition and Brain Sciences Unit<br>Smith, Alicia; University of Cambridge, MRC Cognition and Brain Sciences Unit<br>Kuhn, Isla; University of Cambridge<br>Watkins, Edward<br>Dalgleish, Tim; University of Cambridge, MRC Cognition and Brain Sciences Unit
**Primary Subject Heading**:	Mental health
Secondary Subject Heading:	Patient-centred medicine
Keywords:	Depression & mood disorders < PSYCHIATRY, MENTAL HEALTH, Protocols & guidelines < HEALTH SERVICES ADMINISTRATION & MANAGEMENT, Adult psychiatry < PSYCHIATRY

SCHOLARONE™
Manuscripts

The role of autobiographical memory in patient response to cognitive behavioural therapies for depression: Protocol of an individual patient data meta-analysis

Caitlin Hitchcock *
MRC Cognition and Brain Sciences Unit, University of Cambridge
Caitlin.hitchcock@mrc-cbu.cam.ac.uk

Judita Rudokaite
MRC Cognition and Brain Sciences Unit, University of Cambridge
juditarud@gmail.com

Shivam D Patel
MRC Cognition and Brain Sciences Unit, University of Cambridge
Shivam.patel@mrc-cbu.cam.ac.uk

Alicia Smith
MRC Cognition and Brain Sciences Unit, University of Cambridge
alicia.smith@student.manchester.ac.uk

Isla Kuhn
University of Cambridge
ilk21@cam.ac.uk

Edward Watkins
University of Exeter
E.R.Watkins@exeter.ac.uk

Tim Dalgleish
MRC Cognition and Brain Sciences Unit, University of Cambridge
Cambridgeshire and Peterborough NHS Foundation Trust
Tim.dalgleish@mrc-cbu.cam.ac.uk

* Corresponding author
15 Chaucer Road, Cambridge, CB2 7EF, United Kingdom

For peer review only

For peer review only - http://bmjopen.bmj.com/site/about/guidelines.xhtml

Abstract

Introduction. Cognitive behavioural therapies are one of the most effective treatments for major depression. However, approximately 50% of individuals do not adequately respond to intervention and of those who do remit from a depressive episode, approximately 50% will experience later relapse. Identification of patient-level factors which moderate treatment response may ultimately help to identify cognitive barriers that could be targeted to improve treatment efficacy. This individual patient data meta-analysis explores one such potential moderator - the ability to retrieve specific, detailed memories of the autobiographical past - as cognitive-based therapeutic techniques draw heavily on the ability to use specific autobiographical information to challenge the dysfunctional beliefs which drive depression.

Methods and analysis. We have formed a collaborative network which will contribute known datasets. This will be supplemented by datasets identified through literature searches in Medline, PsycInfo, Web of Science, the Cochrane Central Register of Controlled Trials, and World Health Organisation trials database between December 2018 and February 2019. Inclusion criteria are delivery of a cognitive or cognitive-behavioural therapy for major depression, and measurement of autobiographical memory retrieval at pre-intervention. Primary outcomes are depressive symptoms and clinician-rated diagnostic status at post-intervention, along with autobiographical memory specificity at post-intervention. Secondary outcomes will consider each of these variables at follow-up. All analyses will be completed using random effects models employing restricted maximum likelihood estimation. Risk of bias in included studies will be measured using the Revised Cochrane Risk of Bias tool.

Ethics and Dissemination. The findings will be published in a peer-reviewed journal. Study results will contribute to better understanding of the role of autobiographical memory

For peer review only

in patient response to cognitive behavioural therapies, and may help to inform personalised medicine approaches to treatment of depression.

Registration: PROSPERO CRD42018109673

Strengths and limitations of this study

- This is the first individual patient data (IPD) meta-analysis of the role of autobiographical memory in patient response to cognitive behavioural therapy for major depression.
- This study may identify a patient-level moderator of response to cognitive behavioural therapies.
- Results will advance understanding of whether cognitive behavioural therapies improve this relapse risk factor.
- We anticipate that there may only be a small number of trials which have measured autobiographical memory, which may limit conclusions.

For peer review only

For peer review only - http://bmjopen.bmj.com/site/about/guidelines.xhtml

Introduction

Depression is the second leading cause of disability worldwide[1]. The disorder costs the UK economy an estimated £7.5 billion per year, with this cost expected to increase.[2] Antidepressant medications (e.g., selective serotonin reuptake inhibitors; tricyclic antidepressants) are effective in treating depression,[3-4] but only so long as they are taken.[5-6] Despite efficacious psychological treatments such as interpersonal psychotherapy,[7-8] and cognitive behavioural therapies,[9-10] only 40-60% of NHS patients experience recovery after receiving psychological intervention for depression.[11] Of those who do respond, up to 50% will experience later relapse.[12] Our gold-standard psychological interventions are therefore producing suboptimal outcomes for a significant number of individuals.

Improving treatment success rates is therefore a key priority for social, economic, and health systems. Identification of patient-level factors which may interfere with the efficacy of psychological interventions and predispose an individual toward poorer treatment response may help to explain differential response rates. If we consider cognitive behavioural therapies, which are commonly administered in front-line NHS psychology services (e.g., Improving Access to Psychological Therapies services),[11] identification of cognitive barriers which may impede the ability to complete cognitive therapy tasks may advance our understanding of exactly why some individuals respond poorly, or not at all. Similarly, cognitive predictors of depression which are not shifted by intervention may indicate relapse risk factors.[13] Together, further understanding of patient-level factors that both impede treatment efficacy, and/or are not shifted by treatment and go on to predict relapse may contribute to development of a personalised medicine approach[14] for treatment of depression.

Autobiographical memory, our memory for personal life experiences, is one cognitive factor which plays an important role in the course of depression.[15] In particular, the ability to retrieve specific, detailed memories of single incidents (e.g., *the occasion I met my partner*)

For peer review only - http://bmjopen.bmj.com/site/about/guidelines.xhtml

predicts future depressive symptoms over and above current symptom levels (for a meta-analysis see Sumner et al.).[16] The ability to retrieve specific autobiographical memories not only independently predicts prognosis (e.g., by reducing the ability to solve problems or plan for the future[17-18]), but may also impede the ability to complete CBT for depression.

A core aim of both traditional and later wave CBTs is to change the way that an individual relates to dysfunctional thoughts about the self and world (originally outlined by Beck).[19] Key therapeutic techniques encourage the individual to identify links between thoughts and feelings, by recalling what they said to themselves, how that made them feel, and how that led them to act in a recent situation (e.g., *going to a work function*). For example, a core technique is to encourage the individual to not automatically accept thoughts as facts, but rather, to evaluate the accuracy of each thought (e.g., *no one wants to talk to me*) using specific pieces of evidence recalled from the individual's past (e.g., *last time I was at a work function I had a lovely conversation with a colleague*). The efficacy of such techniques is therefore likely to be influenced by an individual's ability to recall detailed, specific information about their autobiographical past. Treatment manuals instruct cognitive therapists to enrich detail and encourage specificity in patient responses while they are completing such tasks, however, a moderating role of memory specificity in the efficacy of CBTs has yet to be explored.

Similarly, it is possible that this reduction in autobiographical memory specificity may also be improved as a result of completing CBT. In continuously promoting a focus on specific information, and reducing the depressive tendency to overgeneralise information, CBT techniques may actively train an individual to be more specific. Indeed, increasing the specificity of thinking has been previously proposed as a key mechanism of effective CBT for depression.[20] There is some prior literature which suggests that autobiographical memory specificity may improve following a course of cognitive-based intervention, most notably

For peer review only - http://bmjopen.bmj.com/site/about/guidelines.xhtml

CBT[21] and mindfulness-based cognitive therapy.[22] In contrast, evidence that reduced memory specificity persists between depressive episodes[16] may indicate that this relapse risk factor is not commonly shifted by treatment. Determining the impact of CBTs on autobiographical memory specificity, relative to natural recovery, antidepressant medication and other active psychological interventions, will advance our understanding of how treatment impacts this relapse risk factor, and offer important implications for the adjunctive use of autobiographical memory-based interventions (for review see Barry et al.;[23] Hitchcock et al.)[24] to enhance treatment outcomes.

The lack of prior investigation of the interaction between autobiographical memory specificity and CBTs has largely been due to a lack of statistical power to examine moderation effects in prior studies. Prior trials of CBTs which have included a measure of autobiographical memory have recruited small sample sizes (e.g., n=21 in the MBCT group in Williams et al.),[22] and thus been underpowered to explore these effects. Completion of individual patient data meta-analysis (IPD-MA) can overcome this issue. The IPD-MA approach involves synthesis of patient-level data across multiple studies, thereby offering greater statistical potential to explore individual characteristics and how these interact with treatment effects, relative to aggregate meta-analysis which synthesises data at the study-level[25]. We have therefore formed a collaborative network to allow us to complete an IPD-MA evaluating the nature of autobiographical memory specificity in patient response to cognitive behavioural treatment for depression.

Specifically, we will address the following questions:

1) First, does treatment for depression a) induce a change in autobiographical memory specificity? And b) induce a stronger change following CBTs relative to other interventions?

For peer review only

2) Second, does the specificity of autobiographical memory at baseline predict treatment response a) for all interventions (predictor effect); and b) is this effect different for CBTs relative to other interventions (moderation effect)?

Method

Study registration and management

This IPD-MA will be conducted in accordance with PRISMA guidelines,[26] and is registered on PROSPERO, (CRD42018109673). Regular e-mail updates will be used to inform the collaborating network of our activities. Encrypted electronic data sharing clouds and e-mail will be used to exchange data and paperwork between researchers.

Criteria for included studies

Types of studies

Included studies will be both controlled and uncontrolled clinical trials. Studies may be randomised at patient level, cluster-randomised or non-randomised. For cross-over design studies, only data for the initial phase (i.e., pre cross-over) will be used. Articles must be published in English. Unpublished data will be actively sought; hence, non-peer reviewed studies will also be included. Sensitivity analyses will be completed to evaluate the impact of study type on our results.

Participants

Studies must have recruited participants aged 18 years or older. Participants must have a diagnosis of Major Depressive Disorder (MDD) according to the Diagnostic and Statistical Manual of Mental Disorders (any edition) or International Classification of Diseases (any edition) criteria, assessed via a structured clinical interview (e.g., Structured Clinical Interview for DSM Disorders; Mini International Neuropsychiatric Interview). As depression-related deficits in autobiographical memory are evident into remission[16] and National Institute of Health and Care Excellence (NICE) guidelines recommend that CBT

For peer review only - http://bmjopen.bmj.com/site/about/guidelines.xhtml

interventions are used to reduce depressive relapse (e.g., MBCT),[27] participants may either be experiencing a current major depressive episode, or remitted from MDD.

Autobiographical memory

Studies must include an objective measure of the specificity of autobiographical memory. This may be indexed using a cued-recalled task (e.g., the Autobiographical Memory Task),[28] an interview,[29] or sentence completion procedure[30] but responses must be scored for the number of specific, single incident memories retrieved.

Intervention

We will include studies evaluating any psychological therapy for depression which is cognitively-based as per the Beck model (e.g., cognitive therapy, cognitive behavioural therapy, mindfulness-based cognitive therapy). This will be determined by two independent reviewers using the author's intervention description. Interventions may be self-guided or therapist-guided, and delivered in-person or online, and in individual or group-based format. Excluded interventions will be those which directly aim to improve a defined cognitive process or bias (e.g., cognitive/attention/interpretative bias modification, autobiographical memory-based intervention, concreteness training).

Comparison condition

Studies may include no comparison condition (i.e., uncontrolled trials) or may include any type of comparison condition. Comparison conditions are anticipated to be waitlist, any psychosocial intervention (e.g., psychoeducation, counselling), or antidepressant medication. Again, condition type will be determined by two independent reviewers based on the author's intervention description. CBTs will be compared a) against waitlist control (to index effects against natural recovery), b) against all other interventions, c) against other psychological interventions alone and d) against antidepressant medication alone.

Co-primary outcomes

For peer review only - http://bmjopen.bmj.com/site/about/guidelines.xhtml

For Question 1, the primary outcome will be autobiographical memory specificity at post-intervention. This will be operationalised as the proportion of specific, single incident memories reported by the participant during an objective measure of autobiographical memory. Specificity of memories will be rated by researchers (not participants). Proportions (number specific/number of trials) will be calculated for any studies reporting the number of specific memories.

For Question 2, the co-primary outcomes will be treatment response at post-intervention, indexed as a) a score on a continuous measure of depressive symptoms and b) diagnostic status, as rated using a standardised clinician-administered interview. In situations where more than one continuous measure of depression is administered, the Beck Depression Inventory II (BDI-II) will be preferred in analysis as this has been specifically devised to measure changes following CBT interventions. Any studies reporting Beck Depression Inventory I scores will be converted to BDI-II scores using the method outlined in the BDI-II manual.[31]

Secondary outcomes

As it is highly desirable for an intervention to produce stable, lasting change, follow-up assessments will be considered as secondary outcomes. The secondary outcomes for Question 1 will be autobiographical memory specificity (as per defined above) at follow-up assessments. The secondary outcome for Question 2 will be depressive symptoms and diagnostic status (as per defined above) at follow-up assessments.

Follow-up lengths to be included are assessments between one month and two years following the completion of therapy. During analysis, studies including a follow-up assessment between one and three months post-intervention will be grouped to form a short-term follow-up, and any later assessment points will be grouped per six month period (i.e., 6

For peer review only - http://bmjopen.bmj.com/site/about/guidelines.xhtml

months, 12 months). This will result in analysis of follow-up outcomes in the short-term (1-3 months), and at 6, 12, 18, and 24 months post-intervention.

Moderator

For Question 2, the moderating variable will be the proportion of specific, single incident memories reported by the participant during an objective measure of autobiographical memory at baseline.

Search methods for identification of studies

Collaborative network. We have formed a collaborative network of experts in the role of autobiographical memory in depression. Through our own work and knowledge of the field, we have initially identified and obtained access to four individual datasets which provide the data necessary to analyse our research questions. Using this network to identify other potential datasets for inclusion, we will email the autobiographical memory and psychopathology special interest group, and the collaborators of each member of our network. We will also make use of social media such as Twitter to raise awareness of the IPD-MA. This mode of identification is important, as we anticipate that many of the trials which have included an autobiographical memory measure may not list the measure in the method section of the published paper. Indeed, this is the case for the majority of the datasets the collaborative network has already identified.

Electronic searches. As we anticipate that many of the trials which have included an autobiographical memory measure may not list this measure in the published paper, a multi-stage search strategy will be completed in order to identify all potential datasets (see Figure 1).

First, searches will be completed in PsycINFO, Medline, Web of Science, Cochrane database, and WHO trials database, using the string: cognitive therapy[a]

[a] We use the term 'cognitive therapy' although we will also include cognitive behavioural therapies (CBTs) as use of the term 'cognitive behavioural therapy' may miss studies which administer cognitive therapy. Conversely, searches using the term 'cognitive therapy' did identify evaluations of CBTs.

For peer review only - http://bmjopen.bmj.com/site/about/guidelines.xhtml

AND depression AND trial AND any flag words (with OR inserted between every flag word*) we have identified as markers of papers which may potentially include an autobiographical memory measure. These flag words were identified by evaluating common terminology in the abstracts of the four datasets identified through the collaboration network. These terms are: 'mechanism,' 'process', 'memory', 'cognitive function', 'negative thinking', 'rumination', 'depressogenic thinking', 'autobiographical memory'. Searches will be restricted to papers published in or after 1986 as this was the year that the seminal paper on autobiographical memory specificity in depression was published.[28] The full electronic search strategy is available in the Supplementary Materials.

Search results will be imported into Rayyan (a web-based tool for managing systematic reviews) by an administrator (Kuhn) and duplicates will be removed. This will allow for blind screening by two independent reviewers (Rudokaite and Patel). Papers deemed ineligible from title and abstract by both reviewers will be set aside. Full text of the remaining papers will be examined independently by the reviewers to determine eligibility. Any discrepancy in inclusion will be resolved via discussion with Hitchcock, or if necessary, by contacting the study authors for clarification.

Next, a secondary screening will be completed in order to identify studies which may have measured autobiographical memory but not reported on it in the published paper. Through searches in Medline, PsycINFO and Web of Science using the terms autobiographical memory AND depression, we will produce a list of authors who have ever published on autobiographical memory and depression. This list will then be cross-referenced with the results of the primary database search. For studies with an author who has previously published on autobiographical memory, corresponding authors will then be emailed to enquire about unpublished autobiographical memory data.

The electronic search was conducted between December 2018 and February 2019.

For peer review only - http://bmjopen.bmj.com/site/about/guidelines.xhtml

Data collection

Corresponding authors of eligible studies will be emailed to request data. A reminder email will be sent every two weeks. If an author does not respond after 1 month, another author of the study will be contacted. A second attempt to contact this author will follow and so forth until a maximum of three authors are contacted. Study data will be considered unavailable if no study authors have responded to multiple contact attempts, or if authors indicate that they no longer have access to the data.

The authors will be able to supply data via email or an encrypted online data sharing service operated by the University of Cambridge. A single author will be identified for each study to whom all queries about the data collection processes and transformation of individual variables will be addressed. When researchers are cleaning a specific data set they may communicate with the original investigators via telephone discussions or by email.

Data extraction, quality checks, and storage

In accordance with Cochrane recommendations, both published and unpublished measures will be collected for all studies. Individual patient data for specified variables (presented in Table 1) will be extracted into a single dataset. For all outcomes, unimputed and untransformed data will be preferred. Spot checks will be completed to ensure data quality. The pattern of treatment allocation for each included study will be checked to ensure that blinding, randomization and allocation sequence appear appropriate, in accordance with guidelines recommended by Tierney et al.[32] Data will be stored in password protected files on an encrypted University of Cambridge server, and will not include any personally identifiable information.

Table 1. Individual patient data to be extracted from included studies

Trial-level data	Patient characteristics	Outcomes

For peer review only - http://bmjopen.bmj.com/site/about/guidelines.xhtml

Trial identifier	Anonymised participant identifier	Post-treatment self-reported depressive symptoms
Country of completion	Age	Follow-up self-reported depressive symptoms
Type of CBT	Gender	Diagnostic status at post-treatment
Type of comparison group/s	Education history	Diagnostic status at follow-up
Format of treatment (individual, group)	Ethnicity	Post-treatment score for proportion of specific memories
Length of treatment in weeks	Concurrent treatment use (e.g., medication)	Post-treatment score for proportion of non-specific memories
Information about risk of bias	Pre-treatment self-reported depressive symptoms	Follow-up score for proportion of specific memories
Trial design		
	Pre-treatment score for number of specific memories	Follow-up score for proportion of non-specific memories
	Pre-treatment score for number of non-specific memories	
	Any potential cognitive covariates (e.g., rumination, working memory, IQ, executive function)	
	Reasons for missing data	

Risk of bias

For individual studies, risk of bias will be evaluated using the Revised Cochrane Risk of Bias Tool[33] to access study quality and risk of bias due to the randomisation process, deviations from intended interventions, missing outcome data, measurement of the outcome, selection of the reported result and other biases such as conflicts of interest. Using the tool, each study will be rated as of high-, low-, or unclear risk. In order to reduce attrition bias, data will be checked against trial CONSORT diagrams to ensure that data from all randomised participants are included.

For peer review only - http://bmjopen.bmj.com/site/about/guidelines.xhtml

Strategy for data synthesis

Intent-to-treat analysis will be completed using data imputed at within-study level. Between-study heterogeneity will be indexed using the tau statistic. As there are now multiple IPD-MAs establishing the effect of CBTs on depressive outcomes,[34-35] we will proceed directly to evaluating moderation effects.

All analyses will be completed using random effects models employing restricted maximum likelihood estimation. However, if there is considerable heterogeneity in the quality of studies (indexed by the Cochrane Risk of Bias tool) sensitivity analysis will be completed using fixed effects models such that poorer quality studies will not receive equal weighting. If IPD are available for more than seven studies, prediction intervals will be used.[36] If we obtain sufficient power, subsets of studies will be analysed to explore whether the below effects vary based on trauma-history (as trauma exposure may reduce memory specificity),[37] depressive severity, and type of therapy (e.g., MBCT, CBT).

Question 1. The effect of intervention on autobiographical memory specificity will be analysed using an ANCOVA random treatment effect one-stage model with random intercept and memory specificity baseline adjustment with different residual variance per study. Sensitivity analysis will be completed using stratified intercepts.

Question 2.

To determine whether autobiographical memory specificity moderates treatment response, a two-stage model predicting post-treatment depressive symptoms with baseline symptom adjustment is planned to allow synthesis of datasets which used different symptom measures (e.g., BDI-II and Hamilton Depression Rating Scale [HDRS]). The interaction term (baseline score x treatment type) will be estimated per trial, then pooled in a random effects meta-analysis. Sensitivity analysis will next be completed using separate one-stage models

For peer review only - http://bmjopen.bmj.com/site/about/guidelines.xhtml

for different depressive symptom measures. Patient-level covariates will be centred to separate within-trial and across-trial effects.

Effects on diagnostic status will be analysed using a one-stage logistic model with random intercept to account for correlation between the interaction estimate and other parameter estimates. Again, patient-level covariates will be centred to separate within-trial and across-trial effects. Sensitivity analysis will be completed using stratified intercepts. If the one-stage model fails to converge, a two-stage model will be completed.

Ethics and dissemination

Ethics approval was obtained from the Cambridge Psychology Research Ethics Committee (Identifier PRE.2017.100). Contributing studies will be asked to provide evidence of local ethical committee approval and to demonstrate that informed consent has been given to share de-identifed data. Contributing studies will also be required to remove patient identifiers before providing their data. This includes date of birth, which will be converted to age at time of assessment. A data sharing agreement will be signed between the authors of included studies and the IPD-MA research team. The resulting IPD-MA dataset will be shared through the MRC Cognition and Brain Sciences Unit Data Repository (accessed via www.mrc-cbu.cam.ac.uk/publications/opendata/) in accordance with open-science practices.

Patient and Public Involvement: Members of the PPI group formed by individuals with lived experience of depression discussed the project and the role of specificity in CBTs, and will contribute to dissemination of results.

Authors' contributions: CH, TD, & EW designed the project. JR, SDP, AS, & IK contributed to study methods. All authors contributed to writing the paper.

Funding statement: This work was funded by an ESRC grant (Ref: ES/R010781/1) awarded to the first author.

Competing interests statement. None.

For peer review only

For peer review only - http://bmjopen.bmj.com/site/about/guidelines.xhtml

Word count: 3831

Figure 1

Identification of eligible datasets

For peer review only - http://bmjopen.bmj.com/site/about/guidelines.xhtml

1. Ferrari A, Charlson F, Norman R, Patten S, Freedman G, Murray C et al. Burden of Depressive Disorders by Country, Sex, Age, and Year: Findings from the Global Burden of Disease Study 2010. PLoS Medicine. 2013;10(11):e1001547.

2. McCrone P. Paying the price. London: King's Fund; 2008.

3. Fournier J, DeRubeis R, Hollon S, Dimidjian S, Amsterdam J, Shelton R et al. Antidepressant Drug Effects and Depression Severity. JAMA. 2010;303(1):47.

4. Arroll B. Efficacy and Tolerability of Tricyclic Antidepressants and SSRIs Compared With Placebo for Treatment of Depression in Primary Care: A Meta-Analysis. The Annals of Family Medicine. 2005;3(5):449-456.

5. Altshuler L, Kiriakos L, Calcagno J, Goodman R, Gitlin M, Frye M et al. The Impact of Antidepressant Discontinuation Versus Antidepressant Continuation on 1-Year Risk for Relapse of Bipolar Depression. The Journal of Clinical Psychiatry. 2001;62(8):612-616.

6. Altshuler L, Suppes T, Black D, Nolen W, Keck P, Frye M et al. Impact of Antidepressant Discontinuation After Acute Bipolar Depression Remission on Rates of Depressive Relapse at 1-Year Follow-Up. American Journal of Psychiatry. 2003;160(7):1252-1262.

7. de Mello M, de Jesus Mari J, Bacaltchuk J, Verdeli H, Neugebauer R. A systematic review of research findings on the efficacy of interpersonal therapy for depressive disorders. European Archives of Psychiatry and Clinical Neuroscience. 2004;255(2):75-82.

8. O'Hara M, Stuart S, Gorman L, Wenzel A. Efficacy of Interpersonal Psychotherapy for Postpartum Depression. Archives of General Psychiatry. 2000;57(11):1039.

9. Butler A, Chapman J, Forman E, Beck A. The empirical status of cognitive-behavioral therapy: A review of meta-analyses. Clinical Psychology Review. 2006;26(1):17-31.

For peer review only

10. Hofmann S, Asnaani A, Vonk I, Sawyer A, Fang A. The Efficacy of Cognitive Behavioral Therapy: A Review of Meta-analyses. Cognitive Therapy and Research. 2012;36(5):427-440.

11. Pybis J, Saxon D, Hill A, Barkham M. The comparative effectiveness and efficiency of cognitive behaviour therapy and generic counselling in the treatment of depression: Evidence from the 2nd UK National Audit of psychological therapies. BMC Psychiatry. 2017;17(1).

12. Kessler R, Zhao S, Blazer D, Swartz M. Prevalence, correlates, and course of minor depression and major depression in the national comorbidity survey. Journal of Affective Disorders. 1997;45(1-2):19-30.

13. Gotlib I, Joormann J. Cognition and Depression: Current Status and Future Directions. Annual Review of Clinical Psychology. 2010;6(1):285-312.

14. Simon, GE, Perlis R. Personalized Medicine for Depression: Can We Match Patients With Treatments? Am J Psychiatry. 2010;167(12):1445–55.

15. Williams JMG, Barnhofer T, Crane C, Herman D, Raes F, Watkins E, et al. Autobiographical memory specificity and emotional disorder. Vol. 133, Psychological Bulletin. 2007. p. 122–48.

16. Sumner JA, Griffith JW, Mineka S. Overgeneral autobiographical memory as a predictor of the course of depression: A meta-analysis. Behav Res Ther. 2010;48(7):614–25.

17. Jing H, Madore K, Schacter D. Worrying about the future: An episodic specificity induction impacts problem solving, reappraisal, and well-being. Journal of Experimental Psychology: General. 2016;145(4):402-418.

18. Jing H, Madore K, Schacter D. Preparing for what might happen: An episodic specificity induction impacts the generation of alternative future events. Cognition. 2017;169:118-128.

For peer review only

For peer review only - http://bmjopen.bmj.com/site/about/guidelines.xhtml

19. Beck A. Depression: clinical, experimental, and theoretical aspects. London: Staples Press; 1969.

20. Watkins ER. Depressive rumination: Investigating mechanisms to improve cognitive behavioural treatments. Cogn Behav Ther. 2009;38(SUPPL.1):8–14.

21. McBride C, Segal Z, Kennedy S, Gemar M. Changes in Autobiographical Memory Specificity following Cognitive Behavior Therapy and Pharmacotherapy for Major Depression. Psychopathology. 2007;40(3):147-152.

22. Williams JMG, Segal Z V., Teasdale JD, Soulsby J. Mindfulness-based cognitive therapy reduces overgeneral autobiographical memory in formerly depressed patients. J Abnorm Psychol. 2000;109(1):150–5.

23. Barry T, Lenaert B, Hermans D, Raes F, Griffith J. Meta-Analysis of the Association Between Autobiographical Memory Specificity and Exposure to Trauma. Journal of Traumatic Stress. 2018;31(1):35-46.

24. Hitchcock C, Werner-Seidler A, Blackwell S, Dalgleish T. Autobiographical episodic memory-based training for the treatment of mood, anxiety and stress-related disorders: A systematic review and meta-analysis. Clinical Psychology Review. 2017;52:92-107.

25. Fisher D, Copas A, Tierney J, Parmar M. A critical review of methods for the assessment of patient-level interactions in individual participant data meta-analysis of randomized trials, and guidance for practitioners. Journal of Clinical Epidemiology. 2011;64(9):949-967.

26. Moher D, Liberati A, Tetzlaff J, Altman D. Preferred Reporting Items for Systematic Reviews and Meta-Analyses: The PRISMA Statement. PLoS Medicine. 2009;6(7):e1000097.

For peer review only - http://bmjopen.bmj.com/site/about/guidelines.xhtml

27. National Institute of Health and Care Excellence. Depression in adults: recognition and management (NICE Clinical Guidance No. 90). 2009. Retrieved from: https://www.nice.org.uk/guidance/cg90

28. Williams JMG, Broadbent K. Autobiographical Memory in Suicide Attempters. J Abnorm Psychol. 1986;95(2):144–9.

29. Levine B, Svoboda E, Hay J, Winocur G, Moscovitch M. Aging and autobiographical memory: Dissociating episodic from semantic retrieval. Psychology and Aging. 2002;17(4):677-689.

30. Raes F, Hermans D, Williams JMG, Eelen P. A sentence completion procedure as an alternative to the autobiographical memory test for assessing overgeneral memory in non-clinical populations. Memory. 2007;15(5):495–507.

31. Beck A, Steer R, Ball R, Ranieri W. Comparison of Beck Depression Inventories-IA and-II in Psychiatric Outpatients. Journal of Personality Assessment. 1996;67(3):588-597.

32. Tierney JF, Vale C, Riley R, Smith CT, Stewart L, Clarke M, et al. Individual participant data (IPD) metaanalyses of randomised controlled trials: Uidance on their use. PLoS Med. 2015;12(7).

33. Higgins J, Sterne J, Page M, Hróbjartsson A, Boutron I, Reeves B et al. A revised tool for assessing risk of bias in randomized trials In: Chandler, J., McKenzie, J., Boutron, I., Welch, V. (editors). Cochrane Methods Cochrane Database of Systematic Reviews. 2016;(10).

34. Furukawa T, Efthimiou O, Weitz E, Cipriani A, Keller M, Kocsis J et al. Cognitive-Behavioral Analysis System of Psychotherapy, Drug, or Their Combination for Persistent Depressive Disorder: Personalizing the Treatment Choice Using Individual

For peer review only

Participant Data Network Metaregression. Psychotherapy and Psychosomatics. 2018;87(3):140-153.

35. Weitz ES, Hollon SD, Twisk J, Van Straten A, Huibers MJH, David D, et al. Baseline depression severity as moderator of depression outcomes between cognitive behavioral therapy vs pharmacotherapy: An individual patient data meta-analysis. JAMA Psychiatry. 2015;72(11):1102–9.

36. Partlett C, Riley R. Random effects meta-analysis: Coverage performance of 95% confidence and prediction intervals following REML estimation. Statistics in Medicine. 2016;36(2):301-317.

37. Moore S, Zoellner L. Overgeneral autobiographical memory and traumatic events: An evaluative review. Psychological Bulletin. 2007;133(3):419-437.

For peer review only

For peer review only - http://bmjopen.bmj.com/site/about/guidelines.xhtml

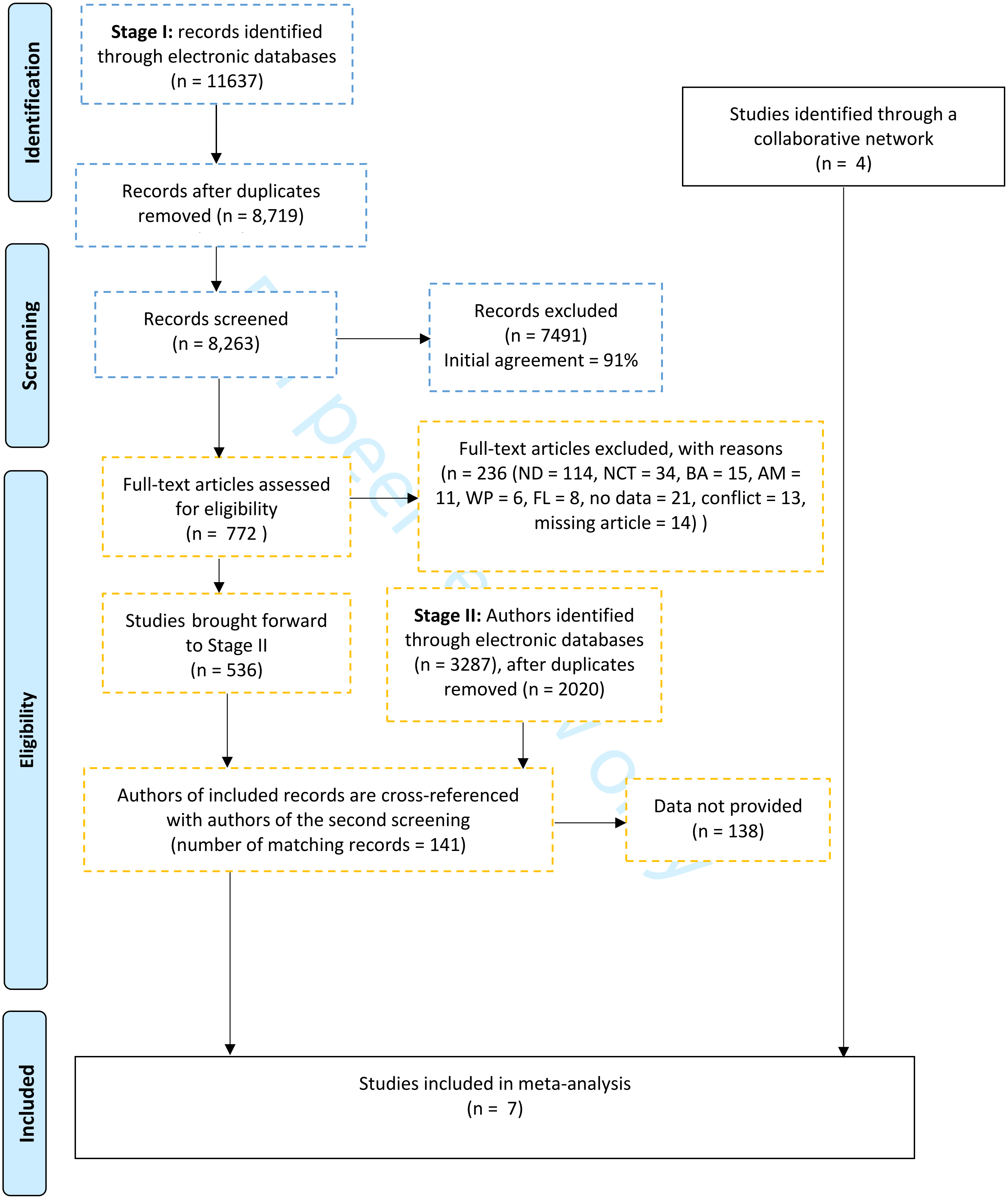
Identification
Screening
Eligibility
Included
Stage I: records identified through electronic databases (n = 11637)
Studies identified through a collaborative network (n = 4)
Records after duplicates removed (n = 8,719)
Records screened (n = 8,263)
Records excluded (n = 7491) Initial agreement = 91%
Full-text articles assessed for eligibility (n = 772)
Full-text articles excluded, with reasons (n = 236 (ND = 114, NCT = 34, BA = 15, AM = 11, WP = 6, FL = 8, no data = 21, conflict = 13, missing article = 14))
Studies brought forward to Stage II (n = 536)
Stage II: Authors identified through electronic databases (n = 3287), after duplicates removed (n = 2020)
Authors of included records are cross-referenced with authors of the second screening (number of matching records = 141)
Data not provided (n = 138)
Studies included in meta-analysis (n = 7)

Overview – Stage 1: CBT & Depression – RCT - keywords

	Hits
Medline	1746
Web of Science	3994
PsycINFO	1189
Cochrane – Trials Database	4566
WHO	149
**Total**	**11637**
**Deduplication**	**8719**

No limits were applied in terms of pu

Medline

Database: Ovid MEDLINE(R) and Epub Ahead of Print, In-Process & Other Non-Indexed Citations, Daily and Versions(R) <1946 to November 5, 2018>
Search Strategy:
----
1 exp behavior therapy/ or (cognit* adj5 behav*).mp. or ((cognit* or behav* or conditioning or relaxation or desensiti*) adj5 (therap* or psychotherap* or train* or retrain* or treat* or modif*)).mp. or (implosive* adj3 therap*).mp.
2 exp depression/ or exp depressive disorder/ or depress*.mp.
3 (Randomized Controlled Trials as Topic/ or randomized controlled trial/ or Random Allocation/ or Double Blind Method/ or Single Blind Method/ or clinical trial/ or clinical trial, phase i.pt. or clinical trial, phase ii.pt. or clinical trial, phase iii.pt. or clinical trial, phase iv.pt. or controlled clinical trial.pt. or randomized controlled trial.pt. or multicenter study.pt. or clinical trial.pt. or exp Clinical Trials as topic/ or (clinical adj trial$).tw. or ((singl$ or doubl$ or treb$ or tripl$) adj (blind$3 or mask$3)).tw. or PLACEBOS/ or placebo$.tw. or randomly allocated.tw. or (allocated adj2 random$).tw.) not (case report.tw. or letter/ or historical article/)
4 (Mechanism* or process* or memor* or (cognitive adj function*) or ((negativ* or depressogenic*) adj3 think*) or ruminat*).mp.
5 1 and 2 and 3 and 4

Web of Science

\# 5 #4 AND #3 AND #2 AND #1
Indexes=SCI-EXPANDED, SSCI, A&HCI, CPCI-S, CPCI-SSH, BKCI-S, BKCI-SSH, ESCI, CCR-EXPANDED, IC Timespan=All years

\# 4 **TOPIC:** ((Mechanism* or process* or memor* or (cognitive near/1 function*) or ((negativ* or depressogenic*) near/3 think*) or ruminat*))
Indexes=SCI-EXPANDED, SSCI, A&HCI, CPCI-S, CPCI-SSH, BKCI-S, BKCI-SSH, ESCI, CCR-EXPANDED, IC Timespan=All years

\# 3 **TOPIC:** (trial* or ((singl* or doubl* or trebl* or tripl*) near/1 (blind* or mask*)) or random*)
Indexes=SCI-EXPANDED, SSCI, A&HCI, CPCI-S, CPCI-SSH, BKCI-S, BKCI-SSH, ESCI, CCR-EXPANDED, IC Timespan=All years

\# 2 **TOPIC:** (depress*)
Indexes=SCI-EXPANDED, SSCI, A&HCI, CPCI-S, CPCI-SSH, BKCI-S, BKCI-SSH, ESCI, CCR-EXPANDED, IC Timespan=All years

\# 1 **TOPIC:** ((cognit* near/5 behav*) or ((cognit* or behav* or conditioning or relaxation

or desensiti*) near/5 (therap* or psychotherap* or train* or retrain* or treat* or modif*)) or (implosive* near/3 therap*))
Indexes=SCI-EXPANDED, SSCI, A&HCI, CPCI-S, CPCI-SSH, BKCI-S, BKCI-SSH, ESCI, CCR-EXPANDED, IC Timespan=All years

PsycINO

#	Query	Results
S12	S4 AND S7 AND S8 AND S11	1,189
S11	S9 OR S10	87,580
S10	(clinical trial*) or ((singl* or doubl* or treb* or tripl*) n1 (blind* or mask*)) or placebo* or (randomly allocated) or (allocated n2 random*)	87,580
S9	(DE "Clinical Trials") OR (DE "Placebo")	15,435
S8	(Mechanism* or process* or memor* or (cognitive function*) or ((negativ* or depressogenic*) n3 think*) or ruminat*)	1,497,463
S7	S5 OR S6	342,107
S6	(DE "Depression (Emotion)") OR (DE "Major Depression")	136,211
S5	depress*	342,107
S4	S1 OR S2 OR S3	228,613
S3	DE "Implosive Therapy"	1,017
S2	DE "Cognitive Therapy"	23,334
S1	(cognit* n5 behav*) or ((cognit* or behav* or conditioning or relaxation or desensiti*) n5 (therap* or psychotherap* or train* or retrain* or treat* or modif*)) or (implosive* n3 therap*)	228,613

For peer review only - http://bmjopen.bmj.com/site/about/guidelines.xhtml

Cochrane

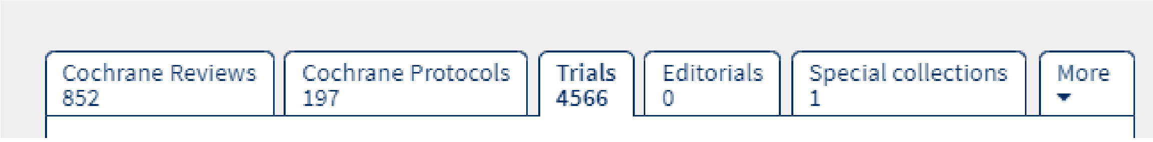

Search Name:
Date Run: 06/11/2018 10:10:46
Comment:

ID Search Hits
#1 ((cognit* near/5 behav*)):ti,ab,kw with Cochrane Library publication date from Nov null to present (Word variations have been searched) 14549
#2 ((cognit* or behav* or conditioning or relaxation or desensiti*) near/5 (therap* or psychotherap* or train* or retrain* or treat* or modif*)) 43009
#3 (implosive* near/3 therap*) 392
#4 MeSH descriptor: [Behavior Therapy] explode all trees 14454
#5 #1 or #2 or #3 or #4 47475
#6 MeSH descriptor: [Depression] explode all trees 9696
#7 MeSH descriptor: [Depressive Disorder] explode all trees 10298
#8 depress* 81488
#9 #6 or #7 or #8 81524
#10 (Mechanism* or process* or memor* or (cognitive function*) or ((negativ* or depressogenic*) near/3 think*) or ruminat*) 131389
#11 #5 and #9 and #10 5640

WHO Trials Registry

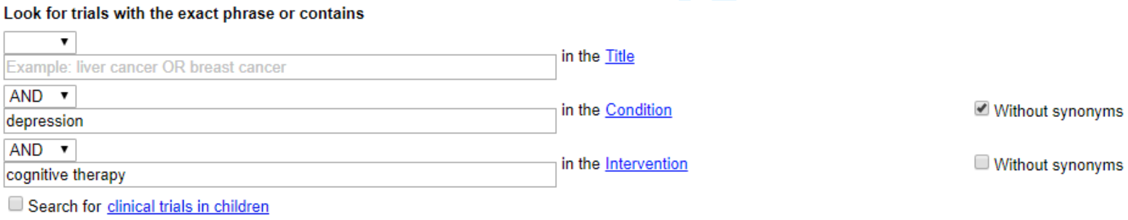

For peer review only - http://bmjopen.bmj.com/site/about/guidelines.xhtml

PRISMA-P (Preferred Reporting Items for Systematic review and Meta-Analysis Protocols) 2015 checklist: recommended items to address in a systematic review protocol*

Section and topic	Item No	Checklist item	(Page No.#)
**ADMINISTRATIVE INFORMATION**			
Title:			
Identification	1a	Identify the report as a protocol of a systematic review	1
Update	1b	If the protocol is for an update of a previous systematic review, identify as such	NA
Registration	2	If registered, provide the name of the registry (such as PROSPERO) and registration number	3
Authors:			
Contact	3a	Provide name, institutional affiliation, e-mail address of all protocol authors; provide physical mailing address of corresponding author	1
Contributions	3b	Describe contributions of protocol authors and identify the guarantor of the review	16
Amendments	4	If the protocol represents an amendment of a previously completed or published protocol, identify as such and list changes; otherwise, state plan for documenting important protocol amendments	NA
Support:			
Sources	5a	Indicate sources of financial or other support for the review	16
Sponsor	5b	Provide name for the review funder and/or sponsor	
Role of sponsor or funder	5c	Describe roles of funder(s), sponsor(s), and/or institution(s), if any, in developing the protocol	
**INTRODUCTION**			
Rationale	6	Describe the rationale for the review in the context of what is already known	4
Objectives	7	Provide an explicit statement of the question(s) the review will address with reference to participants, interventions, comparators, and outcomes (PICO)	7
**METHODS**			
Eligibility criteria	8	Specify the study characteristics (such as PICO, study design, setting, time frame) and report characteristics (such as years considered, language, publication status) to be used as criteria for eligibility for the review	7-10
Information sources	9	Describe all intended information sources (such as electronic databases, contact with study authors, trial registers or other grey literature sources) with planned dates of coverage	10-12
Search strategy	10	Present draft of search strategy to be used for at least one electronic database, including planned limits, such that it could be repeated	11 and supplementary

For peer review only

			materials
Study records:			
Data management	11a	Describe the mechanism(s) that will be used to manage records and data throughout the review	12
Selection process	11b	State the process that will be used for selecting studies (such as two independent reviewers) through each phase of the review (that is, screening, eligibility and inclusion in meta-analysis)	11
Data collection process	11c	Describe planned method of extracting data from reports (such as piloting forms, done independently, in duplicate), any processes for obtaining and confirming data from investigators	12
Data items	12	List and define all variables for which data will be sought (such as PICO items, funding sources), any pre-planned data assumptions and simplifications	Table 1
Outcomes and prioritization	13	List and define all outcomes for which data will be sought, including prioritization of main and additional outcomes, with rationale	9
Risk of bias in individual studies	14	Describe anticipated methods for assessing risk of bias of individual studies, including whether this will be done at the outcome or study level, or both; state how this information will be used in data synthesis	14
Data synthesis	15a	Describe criteria under which study data will be quantitatively synthesised	14
	15b	If data are appropriate for quantitative synthesis, describe planned summary measures, methods of handling data and methods of combining data from studies, including any planned exploration of consistency (such as I^2, Kendall's τ)	
	15c	Describe any proposed additional analyses (such as sensitivity or subgroup analyses, meta-regression)	
	15d	If quantitative synthesis is not appropriate, describe the type of summary planned	
Meta-bias(es)	16	Specify any planned assessment of meta-bias(es) (such as publication bias across studies, selective reporting within studies)	14
Confidence in cumulative evidence	17	Describe how the strength of the body of evidence will be assessed (such as GRADE)	14

*** It is strongly recommended that this checklist be read in conjunction with the PRISMA-P Explanation and Elaboration (cite when available) for important clarification on the items. Amendments to a review protocol should be tracked and dated. The copyright for PRISMA-P (including checklist) is held by the PRISMA-P Group and is distributed under a Creative Commons Attribution Licence 4.0.**

From: Shamseer L, Moher D, Clarke M, Ghersi D, Liberati A, Petticrew M, Shekelle P, Stewart L, PRISMA-P Group. Preferred reporting items for systematic review and meta-analysis protocols (PRISMA-P) 2015: elaboration and explanation. BMJ. 2015 Jan 2;349(jan02 1):g7647.

For peer review only
