# Supplementary material for: Role of autobiographical memory in patient response to cognitive behavioural therapies for depression: protocol of an individual patient data meta-analysis
Source: BMJ Open. 2019 Jun 12;9(6):e031110. doi: 10.1136/bmjopen-2019-031110 (PMC6575822; doi:10.1136/bmjopen-2019-031110)
Supplement: Reviewer comments [file bmjopen-2019-031110.reviewer_comments.pdf]

PEER REVIEW HISTORY

BMJ Open publishes all reviews undertaken for accepted manuscripts. Reviewers are asked to complete a checklist review form (**http://bmjopen.bmj.com/site/about/resources/checklist.pdf**) and are provided with free text boxes to elaborate on their assessment. These free text comments are reproduced below.

ARTICLE DETAILS

**TITLE (PROVISIONAL)**	The role of autobiographical memory in patient response to cognitive behavioural therapies for depression: Protocol of an individual patient data meta-analysis
**AUTHORS**	Hitchcock, Caitlin; Rudokaite, Judita; Patel, Shivam; Smith, Alicia; Kuhn, Isla; Watkins, Edward; Dalgleish, Tim

This article was not externally reviewed at BMJ Open. Protocols that have been independently assessed prior to submission to BMJ Open are usually fast-tracked to publication on the grounds that further substantial changes will not be possible. This independent assessment will usually be external, independent review for both a substantial grant award from a non-commercial or government funder and ethics approval.
